# Integrated metabolome and transcriptome analysis reveals potential mechanism during the bud dormancy transition of *Astragalus membranaceus* (Fisch.) Bge. var. *mongholicus* (Bge.) Hsiao

**DOI:** 10.3389/fpls.2024.1483538

**Published:** 2025-01-21

**Authors:** Huan Guan, Yuhuan Zhao, Qing Chen, Qianqian Zhang, Pengpeng Yang, Shuying Sun, Guilin Chen

**Affiliations:** ^1^ Key Laboratory of Herbage and Endemic Crop Biology, Ministry of Education, School of Life Sciences, Inner Mongolia University, Hohhot, China; ^2^ The Good Agriculture Practice Engineering Technology Research Center of Chinese and Mongolian Medicine in Inner Mongolia, Inner Mongolia University, Hohhot, China; ^3^ Spallation Neutron Source Science Center, Institute of High Energy Physics, Chinese Academy of Science, Dongguan, China

**Keywords:** *Astragalus membranaceus* (Fisch.) Bge. var. *mongholicus* (Bge.) Hsiao, bud dormancy transition, metabolome, transcriptome, co-expression network

## Abstract

*Astragalus membranaceus* (Fisch.) Bge. var. *mongholicus* (Bge.) Hsiao (AMM) is an important medicinal plant that is used for both medicine and food. It is widely used in Asia and South Asia. It is normally cultivated by transplanting the annual rhizomes. Understanding the dormancy of underground buds of AMM is essential for its harvest and transplantation. Despite thorough research on bud dormancy in perennial woody plants, perennial herbs, and especially medicinal plants, such as AMM, have rarely been studied. We analyzed the transcriptome and non-targeted metabolome of dormant buds stage-by-stage to investigate the regulatory mechanism of the transition from endo- to ecodormancy. A total of 1,069 differentially accumulated metabolites (DAMs) participated in amino acid and carbohydrate metabolism. Transcriptome analysis revealed 16,832 differentially expressed genes (DEGs). Functional enrichment analysis indicated that carbohydrate metabolism, hormone signaling pathways, and amino acid metabolism contributed to the transition from endo- to ecodormancy. Starch and sucrose metabolism and hormone signaling pathways were mainly analyzed in the transition between different dormancy states. During the transition from endo- to ecodormancy, the highest content of indole-3-acetic acid (IAA) and the highest number of DEGs enriched in the IAA signaling pathway demonstrated that IAA may play a key role in this process. We obtained candidate genes through co-expression network analysis, such as *BGL*, *GN*, *glgC*, and *glgB*, which are involved in starch and sucrose metabolism. The transcription factors MYB, ERF, bHLH, zinc finger, and MADS-box may regulate the genes involved in hormone signal transduction and starch and sucrose metabolism, which are critical for regulating the transition from endo- to ecodormancy in AMM buds. In summary, these results provide insights into the novel regulatory mechanism of the transition of endo- to ecodormancy in underground buds of AMM and offer new analytical strategies for breaking dormancy in advance and shortening breeding time.

## 1 Introduction

Forming buds and undergoing dormancy are crucial adaptations for perennials during their life cycle to survive harsh winter environments. Environmental factors, such as temperature and light, play a vital role in regulating the induction and release of dormancy (Horvath et al., 2010; Wang et al., 2022a; Rothkegel et al., 2020). Dormancy can be divided into paradormancy, endodormancy, and ecodormancy, depending on the physiological state of dormant tissues (Lang et al., 1987). In the fall, perennial plants enter a dormant period, protecting themselves from harsh winter conditions by temporarily ceasing growth and activity until more favorable conditions return. This process, known as winter dormancy, is essential to the seasonal cycle and supports normal plant growth the following year. Winter dormancy consists of two consecutive stages: endodormancy and ecodormancy (Li et al., 2022b). Endodormancy is mainly regulated by internal factors that inhibit growth, even under favorable environmental conditions (Yordanov et al., 2014; Zhu et al., 2020). Only after the chilling requirements for a specific period, as determined by the variety, are met, leading to the release of endodormancy (Fadón et al., 2020). Insufficient CRs lead to abnormal growth and flowering, resulting in economic loss (Zhang et al., 2017; Ladwig et al., 2019). Once endodormancy is released, bud growth potential is restored and the bud enters an ecodormancy state. However, during this phase, they are sensitive to external environmental factors (Charrier, 2022). When adverse environmental conditions subside, warmer temperatures can lead to visible bud cracking. Studies on woody plants and perennial herbs have shown that bud dormancy is a complex biological phenomenon that requires a series of physiological, biochemical, and molecular mechanisms involving changes in the transport of nutrients, biosynthesis of nucleic acids and proteins, metabolism of carbohydrates, generation of plant endogenous hormones, and other compound shift activities (Horikoshi et al., 2018; Shangguan et al., 2020; André et al., 2022; Li et al., 2022a; Sapkota et al., 2023). Hormone and carbohydrate metabolism may be the main factors that regulate dormancy release (Wang et al., 2018). The regulation of winter dormancy in plants is closely tied to ambient temperature, which means that global warming will significantly affect this process in various ways (Lempe et al., 2024). Therefore, it is essential to understand the stages of dormancy and the mechanisms that regulate them to ensure the sustainable development of production. However, there are few reports on herbaceous plants, particularly medicinal plants.

Plant hormones are considered to be responsible for the regulation of bud dormancy. Abscisic acid (ABA) is involved in the establishment and maintenance of bud dormancy (Chao et al., 2016; Tylewicz et al., 2018). A high ABA content can contribute to maintaining dormancy and can slow down the process of dormancy release (Yang et al., 2021; Li et al., 2018; Horvath et al., 2003). Key genes such as *Nine-cis-polyphenolenoid dioxygenase* (*NCED*) and *ABA 8’-hydroxylase* (*CYP707A*) are responsible for ABA biosynthesis and degradation and are associated with dormancy in perennial plants, such as peaches (Li et al., 2021), *Iris japonica* Thunb (Xu et al., 2022), and petunia (Luo et al., 2023). High ABA levels induce and maintain endodormancy, whereas endodormancy release depends on a sufficient concentration of gibberellin (GA) (Li et al., 2020; Zhang et al., 2018; Gao et al., 2023). GA20 oxidase (GA20ox) and GA3ox are key enzymes that regulate GA biosynthesis, and GA2ox can catalyze the conversion of biologically active GA to non-active forms (Liu and Hou, 2018; Liu et al., 2022). Studies have shown that highly expressed *GA3ox* and *GA20ox* play an important role in regulating bud dormancy release in plants such as tea (Yue et al., 2018) and tree peony (Yuan et al., 2024). IAA and cytokinin (CTK) are signaling molecules that participate in bud dormancy (Qiu et al., 2019; Liu and Sherif, 2019). IAA is one of the most abundant auxins, and the levels of IAA in Chinese fir (*Cunninghamia lanceolata* (Lamb.) Hook) significantly increase during dormancy release (Qiu et al., 2013). IAA levels in underground buds and potato tubers of leafy spurge (*Euphorbia esula* L.) also increased during endodormancy release but decreased during ecodormancy (Chao et al., 2017; Sorce et al., 2000). Studies have shown that the trend of IAA content is similar to that of GA content, indicating that IAA may cooperate with GA to regulate dormancy and promote budbreak (Zhang et al., 2020; Fan et al., 2021). CTK also plays a positive role in the release of bud dormancy (Ito et al., 2021). Hormones may regulate bud dormancy through complex, intertwined pathways (Zhang et al., 2018; Liu and Sherif, 2019); however, the details remain to be further explored.

Carbohydrates have been shown to be closely related to bud dormancy (Park et al., 2009; Khalil-Ur-Rehman et al., 2017; Signorelli et al., 2018). Tarancón et al. (2017) reported that buds were in a state of carbon starvation, induced by both external environmental and endogenous factors during dormancy. Therefore, it is necessary to establish a balance between the stored starch and soluble sugars to maintain their basic functions. Dormancy release is triggered by starch degradation of starch and an increase in soluble sugar content (Zhang et al., 2021a). When *Hippeastrum vittatum* ‘Red Lion’ bulbs and Oriental hybrid lily ‘Sorbonne’ undergo dormancy release, the soluble sugar content increases due to starch degradation (Wang et al., 2014; Liu et al., 2014). Soluble sugars can function as energy supplies or signaling molecules to promote dormancy release. Some studies have confirmed that the interaction between carbohydrates and hormones plays an important role in bud dormancy release (Jiang et al., 2020; Wang et al., 2022a). GA_3_ can significantly enhance the expression of *Sucrose Phosphate Synthase* (*SPS*) during bud dormancy release in *Paeonia lactiflora* (Jiang et al., 2020). The interaction between carbohydrates and hormones mainly focuses on sucrose and IAA during dormancy release in *Polygonatum kingianum* (Wang et al., 2022b). Energy metabolism is also closely related to dormancy in grapes (Rehman et al., 2019) and *Phalaenopsis amabilis* (Chen et al., 2020). Furthermore, glycolysis, tricarboxylic acid (TCA) cycle, and pentose phosphate pathway (PPP) are involved in dormancy release (Zhang et al., 2020). The production of metabolites induced by hydrogen cyanamide (HC) treatment is closely involved in the TCA cycle and PPP, thereby generating sufficient energy to promote endodormancy release (Wang et al., 2021). Similarly, during dormancy release of blueberry flower buds, the expression levels of almost all DEGs involved in the TCA cycle were upregulated, indicating that the energy metabolism process was activated (Li et al., 2022a).

Transcription factors (TFs), including WRKY, APETALA2/ethylene response factors (AP2/ERF), basic region-leucine zipper (bZIP), MADS, Teosinte branched1/Cycloidea/Proliferating cell factor (TCP), and other families are an important group of factors that regulate bud dormancy transition. Previous studies have shown that the WRKY family is an important and conserved TF that plays a crucial role in dormancy release and the cold stress response (Zhang et al., 2017; Shangguan et al., 2020; Zhou et al., 2020). WRKYs and MYBs are upregulated during dormancy release in lily bulbs, indicating that these genes induce bulb dormancy release (Fan et al., 2021). The AP2/ERF family also plays a crucial role in regulating plant growth, development, and freezing stress responses (Yu et al., 2022). The DREB members within the AP2/ERF family are most closely associated with low-temperature stress responses (Liu et al., 2019) and are expressed under the induction of low temperatures (Li et al., 2019b). MADS-box genes are closely related to bud dormancy in various species (Niu et al., 2016), and SOC1 has been shown to affect the dormancy of kiwifruits (Voogd et al., 2015) and poplars (Gómez-Soto et al., 2021).

*Astragalus membranaceus* (Fisch.) Bge. var. *mongholicus* (Bge.) Hsiao (abbreviated as AMM in the following of the manuscript) is a perennial herbaceous medicinal plant that grows in northwest China (Chen et al., 2015). The dried roots of AMM have demonstrated antioxidant, anti-inflammatory, and immunoregulatory functions (Sheik et al., 2021; Zheng et al., 2020) and have a long history of use in traditional Chinese medicine (Dong et al., 2023). It is also one of the most commonly used medicinal materials in South and East Asia (Li et al., 2019a). Additionally, AMM has extensive applications in medicine, food, health products, cosmetics, etc (Ji et al., 2023). Similar to many perennial herbaceous plants, the underground buds on the crowns (the underground part of the stem) of AMM remain dormant during winter to ensure low temperatures. AMM is primarily cultivated on a large scale by transplanting high-quality annual rhizomes in either autumn or spring. Therefore, understanding the dormant period and potential mechanisms for dormancy release of underground buds is crucial for effective transplantation, growth, and harvesting, as well as for reducing breeding cycles. Although research on bud dormancy in perennial woody plants has been relatively thorough, the regulatory mechanism of perennial herbaceous plants, particularly medicinal plants such as AMM, remains not fully understood.

Due to the complexity of genomes and the lack of efficiency in genetic transformation techniques, research on dormancy induction and release in most perennial plants is limited and is a relatively new research field. AMM is an important plant used in traditional Chinese medicine. The underground bud dormancy of AMM hinders the growth and reproduction of these medicinal plants to a certain extent, but the molecular mechanism that regulates dormancy release remains unclear. Through transcriptomics and metabolomics analysis, along with co-expression network analysis, we clarified the changes in genes and metabolites associated with dormancy transition in AMM underground buds. Additionally, we identified molecular pathways and metabolic processes that may be involved in dormancy release. Our findings indicate that hormones and carbohydrate metabolism are crucial in regulating dormancy transition in AMM underground buds. This study confirms that the transition from endo- to ecodormancy in AMM involves the integration of various environmental and internal factors, resulting in a complex regulatory network. Additionally, this study provides new insights into the mechanisms underlying this transition in perennial herbaceous plants.

## 2 Materials and methods

### 2.1 Plant materials

The artificially cultivated annual *Astragalus membranaceus* (Fisch.) Bge. var. *mongholicus* (Bge.) Hsiao (AMM) was introduced from Harqin Banner of Chifeng City (E 118°07′-119°20′, N 41°33′-42°14′) on October 2021, and was identified by Professor Guilin Chen, School of Life Sciences, Inner Mongolia University. Roots with underground buds of AMM were transplanted into pots of 17 cm diameter and 25 cm height with a mixture of soil, peat, and perlite (2:1:1), and carried out near the greenhouse of Inner Mongolia University (E 111°38′, N 40°17′), China.

### 2.2 Experimental design and morphological observation

The underground buds of potted one-year-old AMM (three biological replicates and seven plants per replicate) were transferred to a glasshouse (26°C–15°C day/night, natural light and artificial supplementary lighting, regular watering) every week or so from 31 October 2021. The specific transfer dates were 31 October, 8 November, 15 November, 22 November, 1 December, and 6 December, respectively. The dormancy release phase was assessed based on the method proposed by Wang et al. (2022a) with some modifications. The five morphological indices were observed weekly to evaluate potted plants after being transferred to the glasshouse, including the days until the first plant sprouted in the glasshouse (DFS), the days of bud break percentage ≥60 (DBS), bud break percentage four weeks after being transferred into the glasshouse (BPF), the days until all plants sprouted in the glasshouse (DAS), and plant height four weeks after being transferred into the glasshouse (PHF). The date when most morphological indices showed no significant increase or decrease, even when longer low-temperature exposure occurred, was defined as endodormancy release (Wang et al., 2020). The standard of bud break was when bud scales opened and exposed the leaves. If a bud sprouted on a plant in the pot, it was considered a sprouted plant.

### 2.3 Sample collection and selection

Morphological analysis indicated that the morphological indicators of AMM moved into the greenhouse on 31 October, which showed significant increases or decreases compared with the other groups. This suggested that the plants on this date were in the endodormancy stage. For the plants transferred on 15 and 22 November, most morphological indicators gradually stabilized, leading to the inference that by 22 November, the plants had been released from endodormancy. Once endodormancy breaks, plants enter the ecodormancy stage. Thus, the plants transferred on 1 and 6 December were considered to be in the ecodormancy stage (**Table 1**). Consequently, the samples from 6 December were not included in subsequent research. The potted plants used for sample collection were from batches different from those used for morphological observations. Underground buds were collected on 31 October, 8 November, 15 November, 22 November, and 1 December, respectively (**Supplementary Figure S1**). Since there were at most two large buds on the surface of the crowns, only one plump and one strong bud were collected from each crown. Underground buds were cut, washed with distilled water, and immediately frozen in liquid nitrogen. These samples were stored at −80°C for subsequent transcriptomic, metabolomic, and physiological analyses. Each time point included three biological replicates corresponding to the buds from three distinct plants.

**Table 1:** Morphological observations of AMM after natural chilling treatments in growth room.

Transfer date	DFS (d)	DBS (d)	BPF	DAS (d)	PHF (mm)
31 October 2021	22.33 ± 3.09 a	−^X^	0.24 ± 0.07 c	−^X^	26.67 ± 4.51 d
8 November 2021	17.67 ± 1.25 b	32.00 ± 0.82 a	0.38 ± 0.13 c	36.00 ± 0.82 a	68.14 ± 11.13 cd
15 November 2021	14.00 ± 1.41 bc	23.00 ± 2.83 b	0.76 ± 0.13 b	35.00 ± 2.45 a	157.29 ± 29.63 bc
22 November 2021	13.00 ± 1.63 c	22.00 ± 2.16 bc	1.00 a	25.00 ± 2.94 b	148.24 ± 33.54 ab
1 December 2021	14.00 bc	18.33 ± 1.89 c	1.00 a	25.67 ± 2.05 b	165.48 ± 18.79 a
6 December 2021	13.33 ± 1.70 c	19.00 ± 1.41 bc	1.00 a	24.00 ± 2.45 b	189.07 ± 9.67 a

DFS, the days of the first plant to sprout in the glasshouse; DBS, the days of bud break percentage ≧60; BPF, bud break percentage four weeks after being transferred into glasshouse; DAS, the days of until all plants sprouted in the glasshouse; PHF, plant height four weeks after being transferred into glasshouse. The ‘−^X^’ represented the absence of morphological data, as some plant materials did not germinate due to insufficient cooling requirements. The values were expressed as mean ± standard deviation (SD), different letters represented significant differences (*P <*0.05).

### 2.4 Metabolite extraction and analysis

Metabolites in underground buds collected at five time points were extracted and analyzed by Shanghai Majorbio Bio-Pharm Co., Ltd. (Shanghai, China). The underground buds were crushed, and lyophilized powder (50 mg) was weighed and extracted using 400 µL of 80% aqueous methanol with 0.02 mg/mL L-2-chlorophenylalanin as the internal standard. The mixture was treated with a high-throughput tissue crusher Wonbio-96c (Shanghai Wanbo Biotechnology Co., Ltd.). The supernatant was filtered through a 0.22 μm filter membrane after centrifugation and carefully transferred to sample vials for LC–MS/MS analysis.

Raw data from LC/MS pretreated using Progenesis QI (Waters Corporation, Milford, USA) software, and a three-dimensional data matrix in CSV format was exported. Metabolite annotation was performed according to the main databases: HMDB (http://www.hmdb.ca/) and Metlin (https://metlin.scripps.edu/). Orthogonal partial least squares discriminant analysis (OPLS-DA) and PCA were performed with the R package (v1.6.2). Differentially accumulated metabolites (DAMs) among groups were determined by variable importance in the projection (VIP) >1 (*P <*0.05). These DAMs were further characterized through KEGG functional analysis (http://www.Genome.jp/keg/). K-means clustering of DAMs was performed using SciPy v1.0.0.

### 2.5 Transcriptomic profiling

Total RNA was extracted from 15 underground buds using the EasyPure Plant RNA Kit (TransGen, Beijing, China), according to the manufacturer’s instructions. The RNA quality was assessed using a 2100 Bioanalyzer (Agilent, USA).

RNA purification, reverse transcription, library construction, and sequencing were conducted by Shanghai Majorbio Bio-Pharm Co., Ltd. (Shanghai, China) according to the manufacturer’s instructions (Illumina, San Diego, CA, USA). RNA-seq libraries were sequenced using an Illumina NovaSeq 6000 sequencer (Illumina, San Diego, CA, USA). Raw reads were trimmed and quality controlled using SeqPrep (https://github.com/jstjohn/SeqPrep) and Sickle (https://github.com/najoshi/sickle) with default parameters. Clean reads were used for do *de novo* assembly with Trinity (http://trinityrnaseq.sourceforge.net/). The assembly unigenes were evaluated using BUSCO v3.0.2. The assembled unigenes were annotated using NR (https://www.ncbi.nlm.nih.gov/public/), Swiss-Prot (http://www.uniprot.org/), Pfam (http://pfam.xfam.org/), GO (http://www.geneontology.org), and KEGG (http://www.genome.jp/kegg/) databases. By analyzing the domain information contained in the transcript, gene families of transcription factors were predicted and analyzed using the PlantTFDB5.0 database (http://planttfdb.gao-lab.org/).

Fragments per kilobase of exon per million mapped fragments (FPKM) were used to quantify transcript or gene expression levels. Differential expression analysis between the two groups was performed using the DESeq2 v1.24.0, and DEGs screening criteria were |log2FC|>1 and false discovery rate (FDR) <0.05. FDR was estimated by correcting the *P*-value, and the Benjamini–Hochberg method was used to correct the *P*-value. GO functional enrichment of DEGs was carried out using Goatools v0.6.5, using Fisher’s exact test. KOBAS v2.1.1 was used for enrichment analysis of the DEGs in KEGG pathways based on the Fisher test.

### 2.6 qRT−PCR validation

The relative expression levels of the 16 selected genes were analyzed by qRT−PCR. Primers for candidate genes were designed using Primer-BLAST (https://www.ncbi.nlm.nih.gov/tools/primer-blast/) and are shown in **Supplementary Table S1**. The reference gene was 18S, and all reactions were performed in triplicate using the TransStart Tip Green qPCR SuperMix (TransGen, Beijing, China) on an ABI Prism7500 (Thermo Fisher, Singapore). The thermal cycle of SYBR Green qRT-PCR was as follows: 94°C for 30 s, followed by 40 cycles at 94°C for 30 s, 60°C for 34 s, and 72°C for 30 s. The relative gene expression was calculated using the 2^−ΔΔCt^ method.

### 2.7 Weighted gene co-expression network analysis

After filtering the low-expression genes (mean FPKM of all samples <1), the remaining genes were used for co-expression network analysis with the WGCNA R package (v4.1.3). A hierarchical clustering dendrogram of the genes was created using the dynamic tree-cutting algorithm with mergeCutHeight = 0.25 and minModuleSize = 30. The correlations between gene modules and six sugar contents were analyzed by estimating module–trait relationships. Co-expression networks were formed using Cytoscape software v.3.10.1, with default settings.

### 2.8 Analysis of phytohormones in underground buds

The levels of ABA, GA_3_, IAA, and CTK in underground buds were measured by enzyme-linked immunosorbent assay (ELISA) (Yang et al., 2001) using an ELISA kit (MLBIO, Shanghai, China), repeated three times. Approximately 50 mg of buds was ground and extracted in 1 mL of cold 80%(v/v) methanol overnight at 4°C. After centrifugation for 10 min at 10,000*×g* and 4°C, the supernatant was collected. An additional 500 μL of 80% methanol was added to the precipitate for further extraction, which was performed for 2 h at 4°C. Following another round of centrifugation for 10 min at 10,000*×g* and 4°C, the supernatants were combined and dried with nitrogen. The pellet was then dissolved in 300 μL of 30% methanol and filtered through a filter membrane (0.22 µm). Hormone levels were determined according to the manufacturer’s instructions using an ELISA kit.

### 2.9 Carbohydrate concentration measurements

The soluble sugar and sucrose contents in the underground buds were determined using the approach of Du et al. (2020). Starch was extracted and quantified using a modified method described by Zhang et al. (2022).

### 2.10 Statistical analysis

SPSS software v19.0 (SPSS, Inc., Chicago, IL, USA) was used to conduct one-way analysis of the data with Duncan’s multiple comparison test. The results are expressed as mean ± standard deviation (SD) of three biological replicates. Line charts were generated using GraphPad Prism v9.0.

## 3 Results

### 3.1 Determining the dormancy status and physiological dynamics of underground buds of AMM

We followed the method used by Wang et al. (2022a) to determine the dormant states of *P. lactiflora*. Using this method, we transferred the one-year-old *Astragalus membranaceus* (Fisch.) Bge. Var. *mongholicus* (Bge.) Hsiao (AMM) under natural chilling conditions into a greenhouse on 31 October, 8 November, 15 November, 22 November, 1 December, and 6 December 2021, respectively, to monitor the development of underground buds. The bud break percentage four weeks after being transferred into the glasshouse (BPF) on 31 October was only 24%, and its BPF never reached 60% (**Table 1**). The days of the first plant to sprout in the glasshouse (DFS), the days of bud break percentage≧60 (DBS), and the days required for all plants to sprout in the glasshouse (DAS) gradually decreased. The BPF increased significantly on 15 November and reached 100% by 22 November; most of the indices were also stable. Based on these observations, 22 November was determined as the endodormancy release date. 1 and 6 December were classified as the ecodormancy stages. Because the samples collected on 1 and 6 December were in the same dormant stage, only the samples from the first five time points were selected for further analysis, and they were named 31 October, 8 November, 15 November, 22 November, and 1 December, respectively.

**Figure 1:**
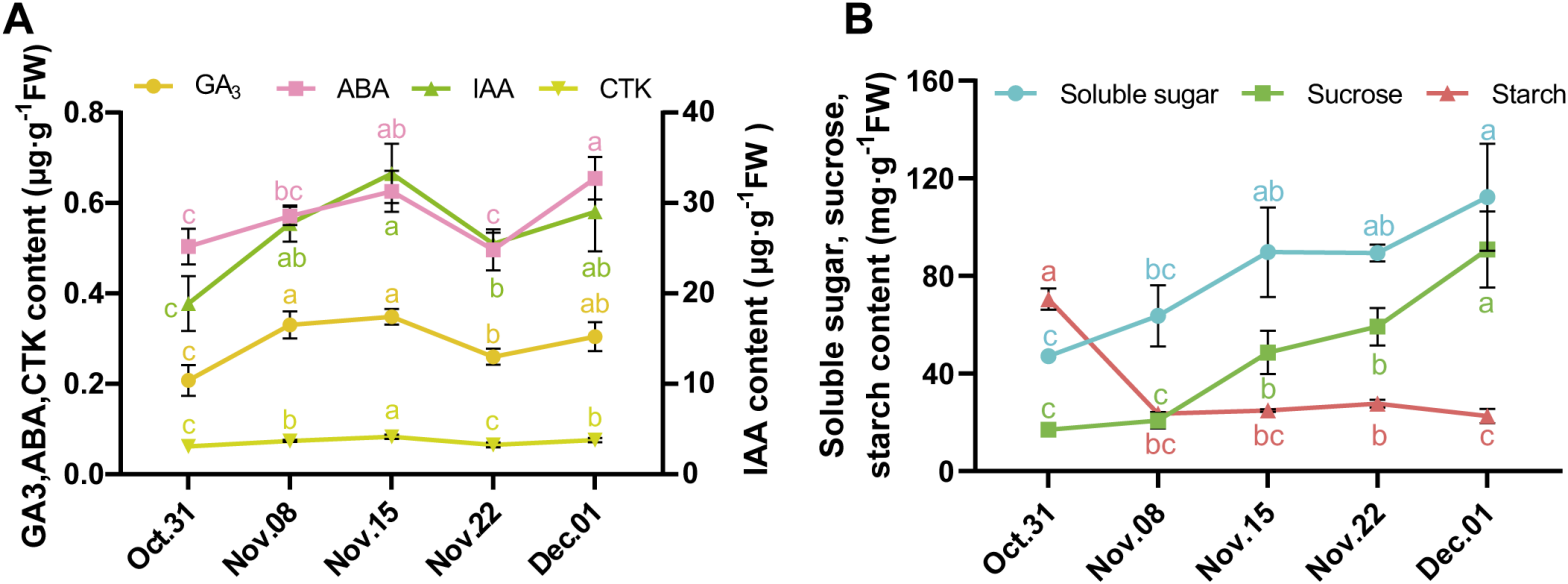
Changes of hormone and sugar content in AMM underground buds during the transition from endo- to ecodormancy. **(A)** The contents of GA_3_, ABA, IAA, and CTK in the underground buds of AMM during different dormant stages. **(B)** Changes in the contents of soluble sugar, sucrose, and starch in AMM underground buds during the transition from endo- to ecodormancy.

Plant hormones play an important role in the dormancy transition process; therefore, we measured hormone levels in underground buds at five time points. IAA had the highest concentration, exceeding 20 μg·g^−1^ FW, whereas CTK had the lowest concentration, recorded at just over 0.06 μg·g^−1^ FW (**Figure 1A**). The trends in the levels of the four hormones were similar: they gradually increased from 31 October to 15 November, reaching a peak on 15 November. Following this peak, the levels decreased sharply on 22 November, before experiencing a significant increase again.

Changes in carbohydrate levels are associated with dormancy and are often used as markers to divide bud dormancy. In underground buds, the contents of soluble sugar and sucrose consistently increased from 31 October to 1 December. However, starch levels sharply declined from 31 October to 8 November. Similarly, significant changes were observed in morphological indices, such as DFS, DBS, and DAS, during this period. On 1 December, the starch content decreased (**Figure 1B**). In conclusion, based on the morphological indices and trend in starch content, 31 October was divided into the endodormancy stage. The period from 31 October to 22 November was identified as the endodormancy release stage, and the ecodormancy stage was subsequently established.

### 3.2 Identifying the transition from endo- to ecodormancy of AMM underground buds by metabolic differences

**Figure 2:**
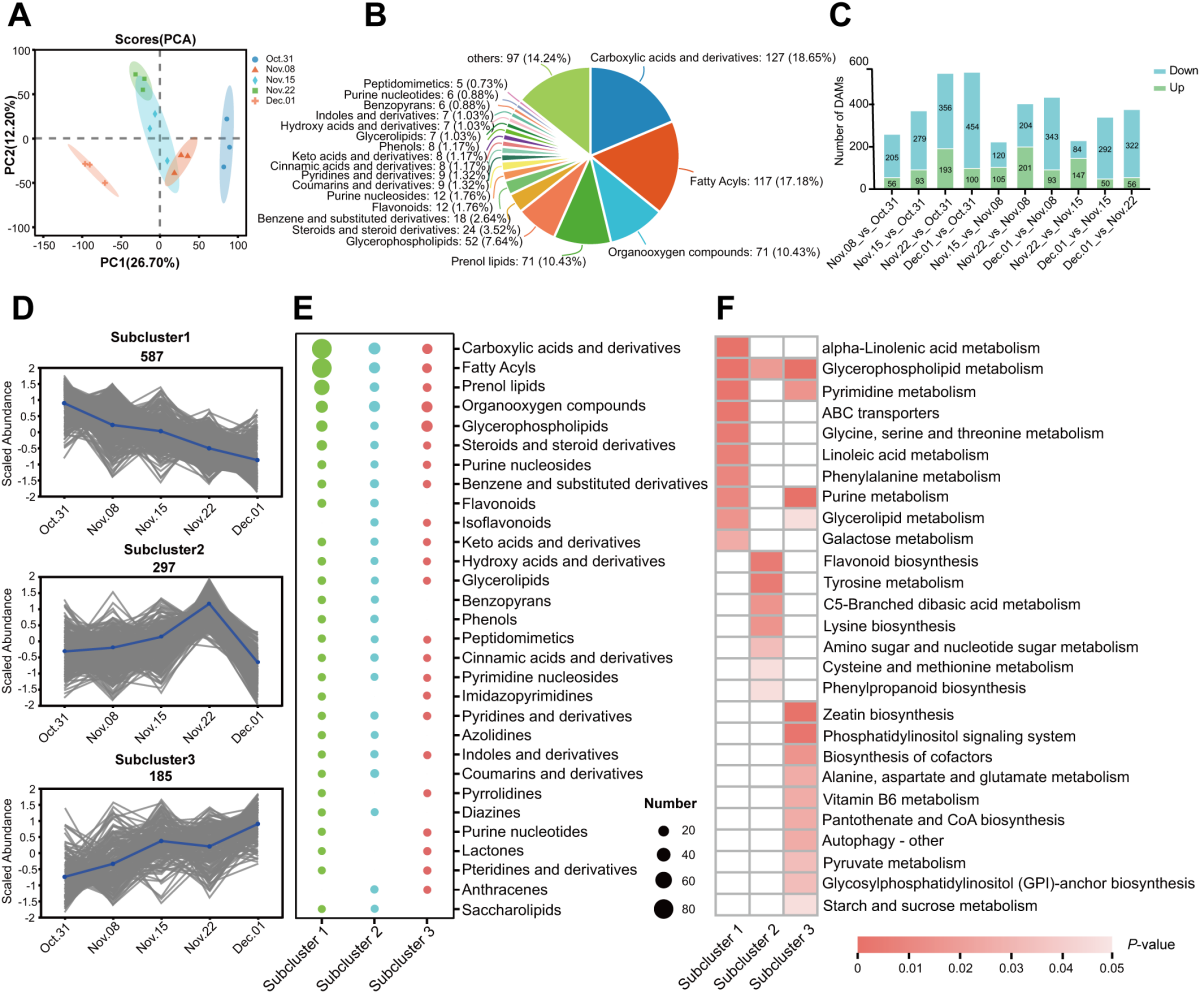
Metabolomic analysis of the transition from endo- to ecodormancy in underground buds of AMM. **(A)** Principal component analysis (PCA) of metabolome data in different groups; **(B)** Classification and number of 1,069 DAMs; **(C)** Statistical analyses of the number of DAMs from different pairwise comparisons. **(D)** K-means cluster analysis of DAMs. **(E)** The number of different classes of DAMs in different subclusters. **(F)** KEGG enrichment analysis of DAMs in the three subclusters.

To understand the metabolic profiles of AMM underground buds during the transition from endo-to ecodormancy, 15 samples from five transfer time points were collected and subjected to non-targeted metabolomic analysis using LC–MS/MS. In total, 1,816 metabolites were identified. Principal component analysis (PCA) showed that 31 October and 1 December were separated from the other samples (**Figure 2A**), indicating that the accumulation of metabolites was associated with different dormancy stages. After pairwise comparison, 1,069 DAMs were obtained, consisting of 21 classes, including 127 carboxylic acids and derivatives,117 fatty acyls, 71 organooxygen compounds, 71 prenol lipids, 52 glycerophospholipids, 24 steroids and steroid derivatives, 18 benzene and substituted derivatives, 12 flavonoids, 12 purine nucleosides, and 171 compounds from other classes (**Supplementary Table S2**; **Figure 2B**). After pairwise comparison, it was found that the highest number of DAMs were observed in the comparison of 1 December_vs_31 October, with 454 downregulated and 100 upregulated metabolites. This was followed by the 22 November_vs_31 October, which showed 356 downregulated and 193 upregulated metabolites. Additionally, a large number of DAMs were identified on 1 December_vs_ 22 November. These findings suggest a significant change in the metabolites of underground buds on 31 October, 22 November, and 1 December, which may indicate different dormancy states at these three time points (**Figure 2C**). During the endodormancy release in the underground buds of AMM, we analyzed 1,069 annotated DAMs using K-means cluster analysis to track changes in their relative abundance. The DAMs were divided into three subclusters (**Figure 2D**). Based on the accumulation patterns of the subclusters, we observed that the metabolites in subcluster 1 showed a continuously decreasing trend during the transition from endo- to ecodormancy (from 31 October to 1 December), with relatively high numbers of carboxylic acids and derivatives and fatty acyls, accounting for 21.3% and 21.04% of the total, respectively (**Figure 2E**; **Supplementary Table S3**). In contrast, the metabolites in subcluster 3 showed a gradually increasing trend during the transition from endo- to ecodormancy, with glycerophoripids, organooxygen compounds, carboxylic acids and derivatives, and fatty acyls dominating. The metabolites in subcluster 2 exhibited continuous accumulation during endodormancy release (from 31 October 31 to 22 November), with carboxylic acids and derivatives, fatty acyls, and organooxygen compounds being the most prevalent, accounting for 15.14%, 12.43%, and 11.89%, respectively.

KEGG enrichment analysis showed that the clustered DAMs were involved in different metabolic and biosynthetic pathways (**Figure 2F**). The darker the color, the smaller is the *P*-value. During the transition from endo- to ecodormancy, DAMs are enriched in various amino acid metabolism pathways. In addition, the metabolic pathways of alpha-linolenic acid, glycerophospholipid, glycine, serine, threonine, phenylalanine, and galactose were weakened (subcluster 1). However, glycerophospholipid metabolism, zeatin biosynthesis, and starch and sucrose metabolism increased during this process (subcluster 3). The metabolites involved in flavonoid biosynthesis, tyrosine metabolism, and lysine biosynthesis were enriched during endodormancy release, indicating that these pathways were activated when endodormancy was released (subcluster 2).

### 3.3 Identifying the differently expressed genes in underground buds of AMM

**Figure 3:**
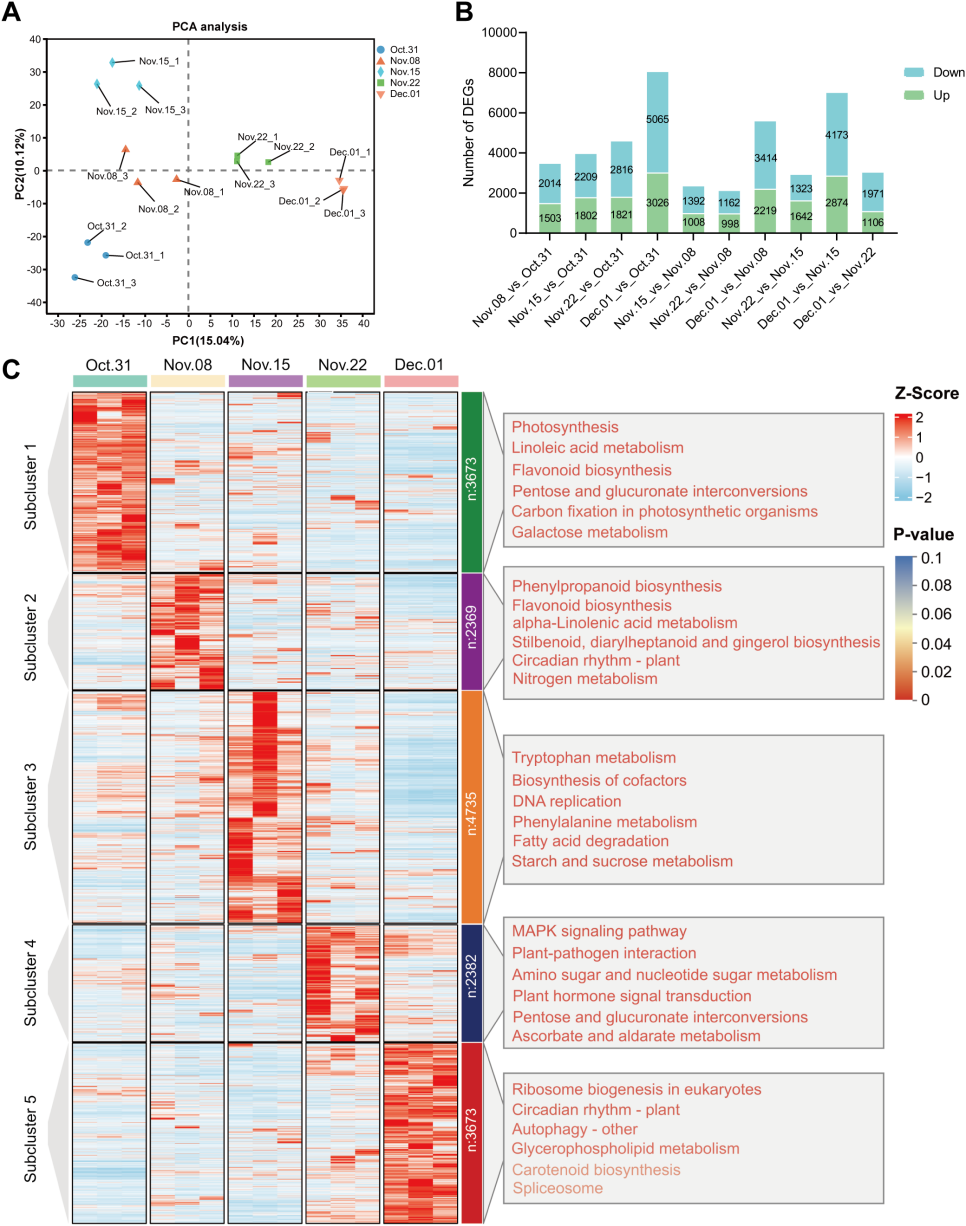
Analysis of DEGs in underground buds of AMM during the transition from endo- to ecodormancy. **(A)** Principal component analysis PCA for transcriptomes; **(B)** Bar graph of up- and downregulated DEGs from different pairwise comparisons; **(C)** K-means cluster analysis and KEGG enrichment analysis of 16832 DEGs. The DEGs are divided into five subclusters. The top 6 KEGG pathways ranked by *P*-value in each subcluster are listed on the corresponding right panel. The font color represents the *P*-value, and the redder the color, the smaller the *P*-value.

As shown in **Supplementary Table S4**, the assembled unigenes were evaluated, revealing an N50 length of 1,598 bp and a BUSCO score of 65.7%. The transcriptome sequencing results showed a total of 43,271,254–72,963,680 clean reads with Q30 ranging from 91.36% to 92.82%, and a GC content of 43% in the AMM underground buds. The results showed that 72.31% to 75.14% of the clean reads could be mapped to the assembled reference sequence (**Supplementary Table S5**), which provided available transcript information for analysis. PCA of the transcriptome data revealed that the three biological repeats in each group were highly similar. Additionally, 31 October, 15 November, and 1 December were significantly different from those of the other groups. The first principal component (PC1) accounted for 15.04% of the total variance, separating 31 October, 8 November, and 15 November from 22 November and 1 December (**Figure 3A**). 1 December_vs_31 October had the largest number of DEGs, followed by 1 December_vs_15 November in which 3,026 and 2,874 were upregulated, and 5,065 and 3,173 downregulated DEGs were identified, respectively (**Figure 3B**). Unlike the transcriptome results, there were more DAMs on 1 December_vs_22 November, indicating significant changes in gene expression on 15 November, possibly in preparation for metabolite accumulation on 22 November. To investigate gene expression patterns during different dormancy stages, DEGs were further divided into five subclusters using K-means clustering analysis based on their relative expression, which was not the same as the clusters from the metabolic analysis. The gene expression levels in subclusters 1–5 peaked on 31 October, 8 November, 15 November, 22 November, and 1 December, respectively, which corresponded to the endodormancy stage, endodormancy release stage, early stage of complete release of endodormancy, endodormancy release date, and ecodormancy stage, respectively (**Figure 3C**).

To further determine the biological functions of the DEGs in the five subclusters, we performed GO and KEGG enrichment analyses. GO analysis of these subclusters showed that DEGs were enriched in molecular function, cellular component, and biological processes (**Supplementary Table S6**). Genes highly expressed during endodormancy were enriched in linoleic acid metabolism, flavonoid biosynthesis, and plant hormone signal transduction (subcluster 1). The genes in subcluster 2 were enriched in phenylpropanoid biosynthesis, flavonoid biosynthesis, and plant hormone signal transduction. The genes in subcluster 3 were enriched for tryptophan metabolism, DNA replication, and starch and sucrose metabolism. The KEGG pathways enriched by the DEGs in subcluster 4 included plant hormone signal transduction, amino sugar and nucleotide sugar metabolism, and phenylpropanoid biosynthesis, consistent with those observed in the metabolome analysis. Genes highly expressed during the ecodormancy phase were enriched in ribosome biogenesis in eukaryotes, circadian rhythm, autophagy, and glycerophospholipid metabolism (**Figure 3C**; **Supplementary Table S6**).

### 3.4 Validating DEGs by qRT-PCR

To validate the results of the transcriptomic analysis, we carried out qRT-PCR on 16 selected genes, which were mainly involved in hormone biosynthesis, signal transduction, starch and sucrose metabolism, and TFs (**Supplementary Table S7**). The results indicated that *GA3* (TRINITY_DN34977_c0_g1), *GID2* (TRINITY_DN18555_c0_g1), and *ARF7* (TRINITY_DN6952_c1_g2) were upregulated and then downregulated during endodormancy release, whereas *GA2ox2* (TRINITY_DN8660_c0_g1) and *SAUR32* (TRINITY_DN10943_c0_g1) were downregulated (**Supplementary Figure S2**). Overall, the qRT-PCR results were consistent with the transcriptomic results, indicating the reliability of the RNA-sequencing data.

### 3.5 The responses of hormone signal transduction related genes during the dormancy transition

According to KEGG analysis, the genes involved in hormone signal transduction were significantly enriched during endodormancy release (**Figure 3C**). The profiles of the DEGs involved in hormone biosynthesis and metabolism were investigated (**Supplementary Table S8**). The key DEGs involved in ABA, GA, and IAA synthesis, such as *NCEDs*, *GA20ox*, *GA3ox*, *YUCCA2*, and *YUCCA10*, were highly upregulated from 31 October to 15 November and significantly decreased from 22 November to 1 December (**Supplementary Figures S3A–C**). Five ABA degradation genes, *CYP707As* were identified, in which the expression levels of three *CYP707A2* genes were relatively low from 31 October to 15 November but increased significantly from 22 November to 1 December. Among the genes related to CTK metabolism, three *CKXs* (*CKX1*, *CKX5*, and *CKX6*) were downregulated from 31 October to 15 November (**Supplementary Figure S3D**), consistent with the changes observed in GA, ABA, IAA, and CTK contents.

**Figure 4:**
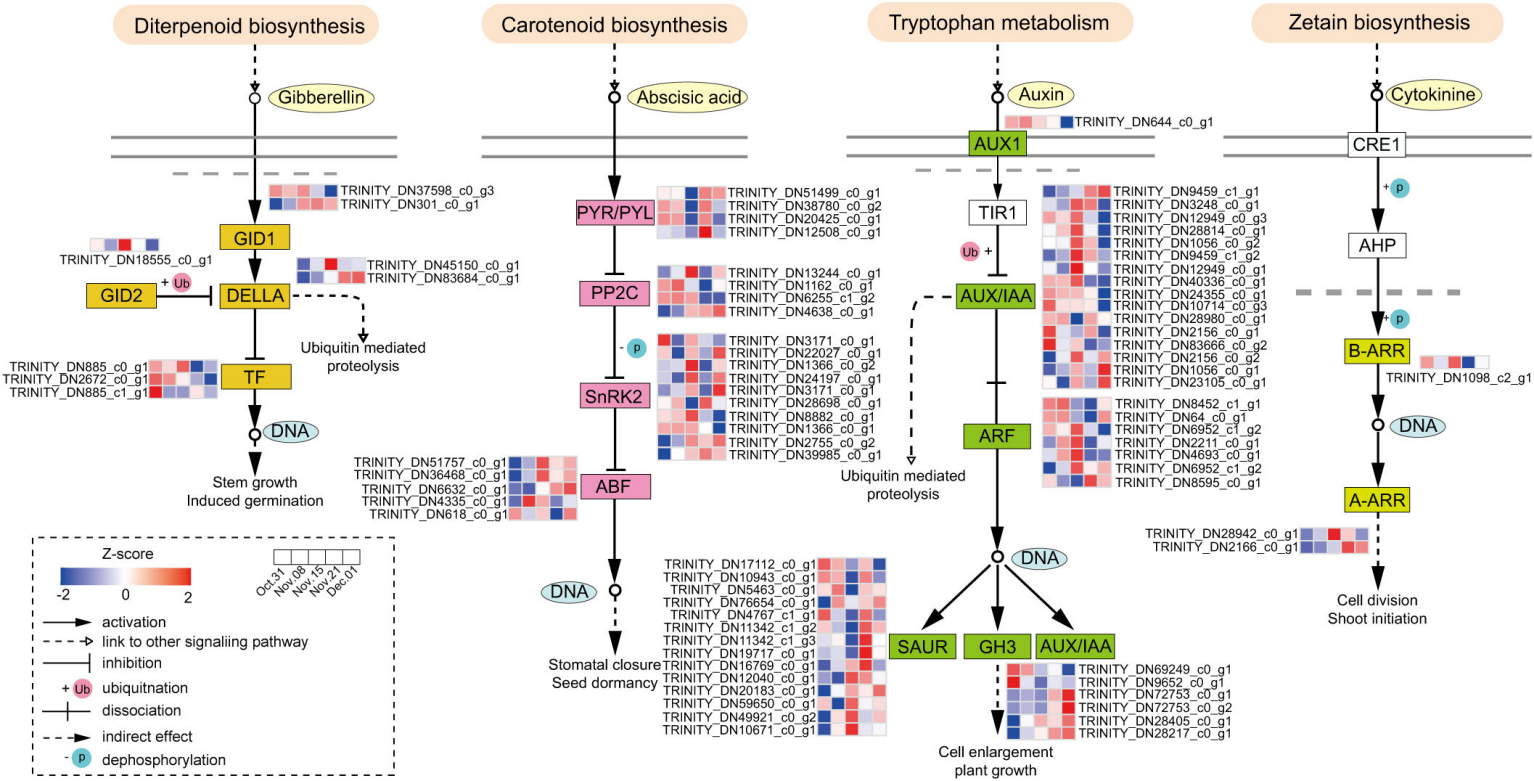
Hormone signaling pathways during the transition from endo- to ecodormancy in the underground buds.

To explore the role of hormone signal transduction in the transition from endo- to ecodormancy, we analyzed the genes in subclusters 1, 4, and 5. These subclusters exhibited high expression during the endodormancy stage, at the endodormancy release date, and during the ecodormancy stage. Additionally, we discovered that hormone levels peaked on 15 November, coinciding with the highest number of DEGs in the samples collected at this time. Therefore, we analyzed the genes in subcluster 3. A total of 78 DEGs related to ABA, GA, IAA, and CTK signaling were identified (**Supplementary Table S9**). There were more DEGs involved in IAA and ABA signaling pathways, 44 and 23, respectively (**Figure 4**). ABA-related genes *PYLs*, were downregulated from 31 October to 15 November. In contrast, *SNRK2s* and *ABFs* were significantly upregulated during the same period. DEGs associated with *AUX/IAA* and *ARFs* were upregulated gradually from 31 October to 15 November, peaked on 15 November, and then decreased significantly, whereas *SAUR* and *GH3* displayed the opposite trend. Fewer genes were involved in GA and CTK signaling and *GID2* was expressed significantly from 31 October to 15 November, followed by a significant decrease. Among the three annotated PIFs, the expression level of *PIF3* was highest on 15 November. The expression patterns of genes in the CTK signaling pathway also varied, with two *ARR-As* gradually upregulated during the transition from endo- to ecodormancy.

**Figure 5:**
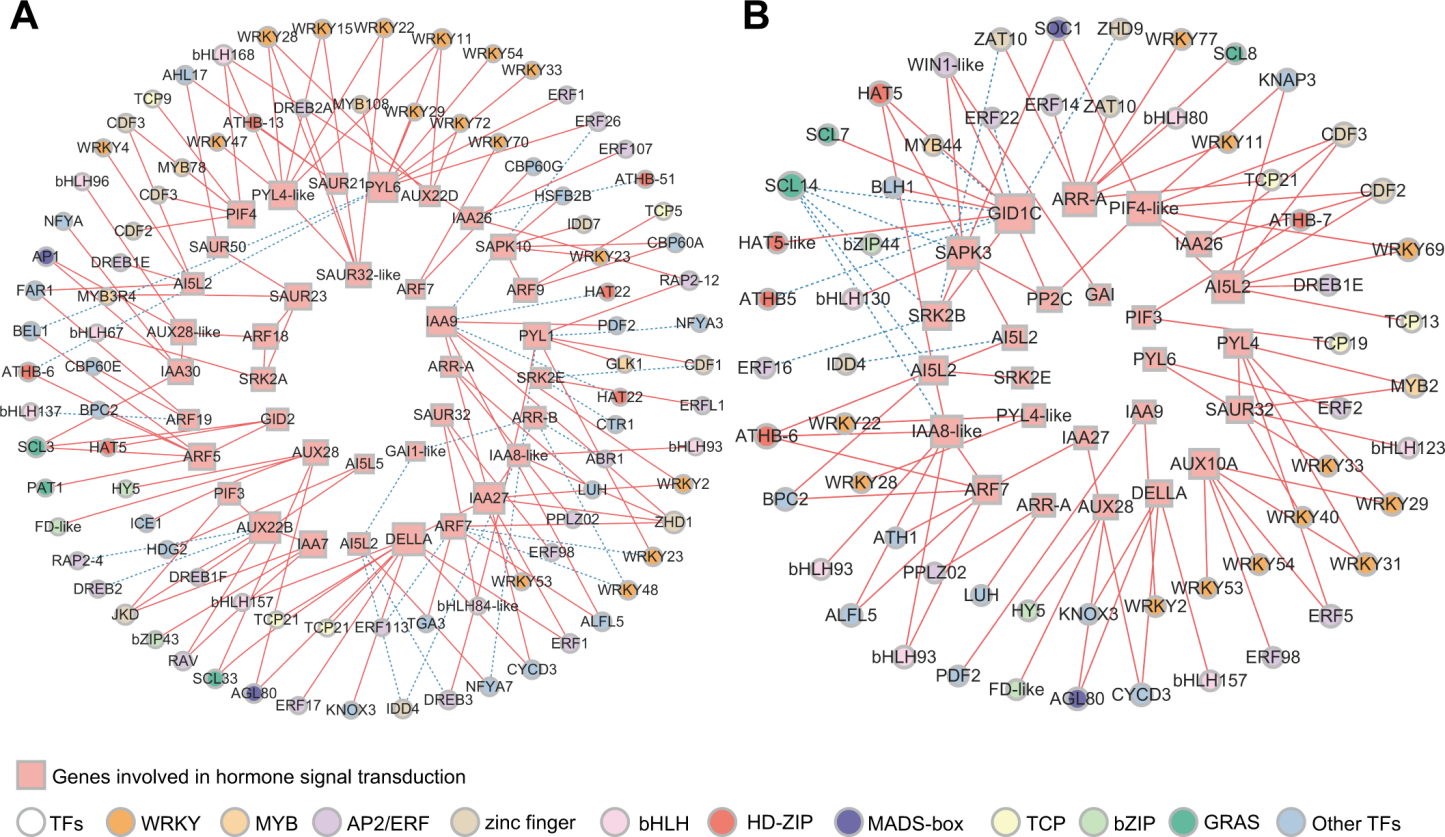
Co-expression network analysis of genes involved in hormone signal transduction and TFs. **(A)** Correlation network of structural genes involved in GA, ABA, IAA, and CTK signaling pathways and TFs in KEGG analysis during the process of endodormancy release. **(B)** Correlation network of structural genes involved in GA, ABA, IAA, and CTK signaling pathways and TFs in KEGG analysis during the transition from endodormancy to ecodormancy (*r* >0.9 or *r* <−0.9, *P* <0.05). The size of rectangles and circles indicates the degree of connectivity. The greater the degree of connectivity, the larger the shapes. The solid red line represents positive correlation and the dashed blue line represents negative correlation.

To identify the key genes involved in hormone signal transduction and the potential TFs regulating this pathway during dormancy release, we performed co-expression network analysis of DEGs involved in hormone signal transduction and differentially expressed TFs. In total, 187 differentially expressed TFs were identified within subclusters 1, 3, 4, and 5. Cytoscape was used to visualize the results (*r >*0.9 or <−0.9, *P <*0.05, **Figure 5**). The genes involved in the IAA and ABA signaling pathways were activated from 31 October to 22 November (**Figure 5A**). According to the gene connectivity, the top three genes in IAA signaling pathway were *AUX22B* (TRINITY_DN2156_c0_g1), *IAA9* (TRINITY_DN12949_c0_g1), and *IAA27* (TRINITY_DN1056_c0_g2). The top three genes in the ABA signaling pathway were *PYL6* (TRINITY_DN51499_c0_g1), *PYL4-like* (TRINITY_DN38780_c0_g2), and *PYL1* (TRINITY_DN20425_c0_g1). Additionally, *DELLA* (TRINITY_DN45150_c0_g1) in the GA signaling pathway also had a high level of connectivity. The AP2/ERF (18), WRKY (16), and zinc finger (8) families account for a large number of genes involved in hormone signal transduction. Among them, *WRKY11* and *WRKY28* within the WRKY family showed the same number of interaction pairs, and WRKY family genes were mainly co-expressed with IAA and ABA signaling pathway genes.

During the transition from endo- to ecodormancy, *GID1C* (TRINITY_DN37598_c0_g3), *PIF4-like* (TRINITY_DN2672_c0_g1), *ARR-A* (TRINITY_DN2166_c0_g1), *SAPK3* (TRINITY_DN3171_c0_g2), *AI5L2* (TRINITY_DN6632_c0_g1), and *IAA8-like* (TRINITY_DN12949_c0_g3) were highly correlated (**Figure 5B**). However, *GID1C* did not show high connectivity during endodormancy release (**Figure 5A**). This result suggests that genes from the same hormone signaling pathway may function at different stages of the transition from endo- to ecodormancy. Furthermore, WRKY, AP2/ERF, bHLH, and zinc finger were the key TF families regulating hormone signal transduction, with 12, 9, 6, and 6 annotated genes, respectively.

### 3.6 Analyzing the weighted gene correlation network

**Figure 6:**
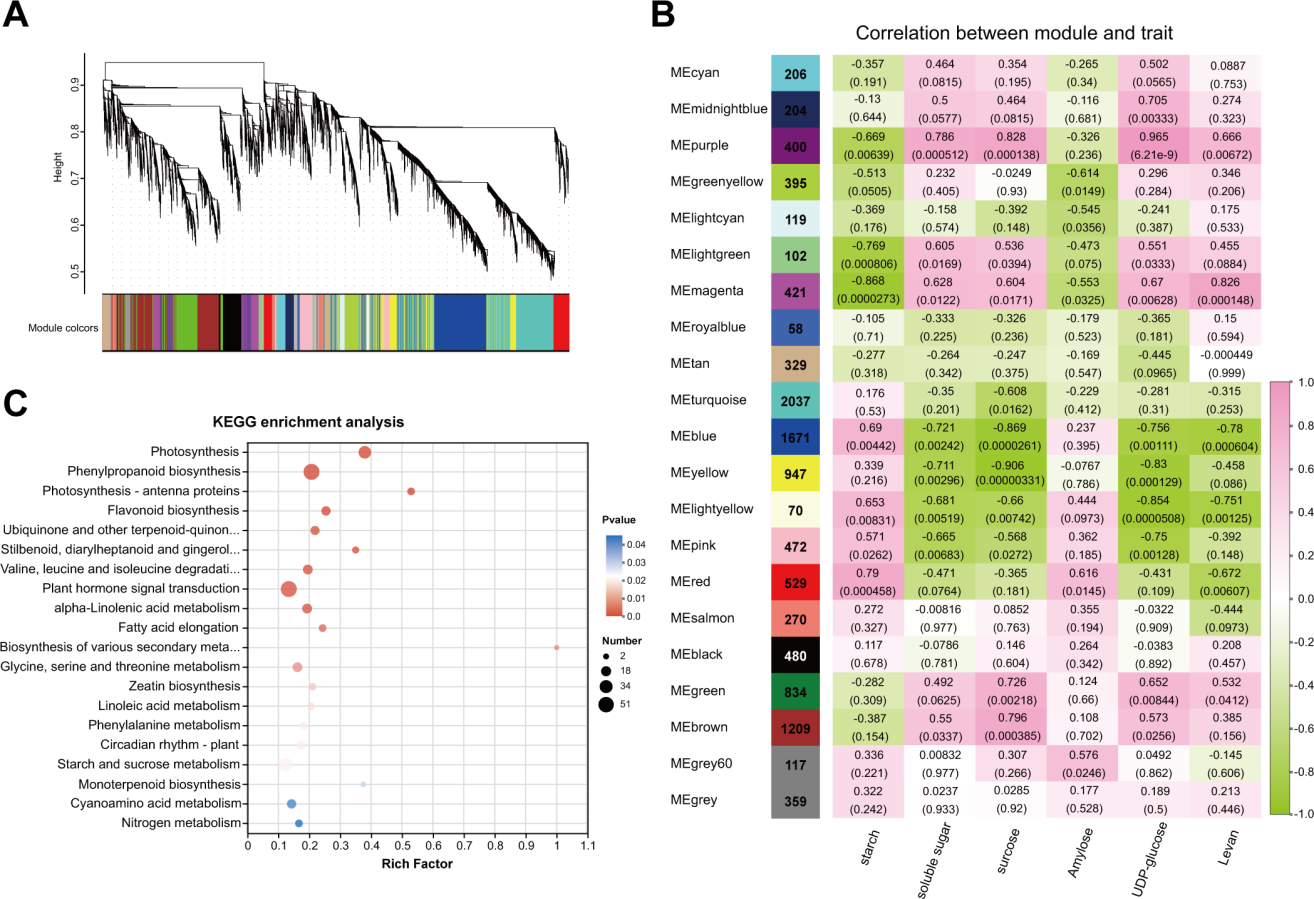
WGCNA analysis of transcriptome data during dormancy transition in underground buds of AMM. **(A)** Dendrogram showing the co-expression modules of 16832 DEGs identified during the dormant phase transition through WGCNA, with the major tree branches consisting of 21 modules labeled with different colors. **(B)** Heat map showing the correlation between modules and metabolites in starch and sucrose metabolism pathways. **(C)** Top 20 of KEGG enrichment analysis of DEGs in the co-expression modules related to the sugar contents. Each column corresponds to a time point and each row corresponds to a module labeled with different colors. Pink and green represent positive and negative correlations, respectively.

Metabolomic and transcriptomic analyses indicated that starch and sucrose metabolism was enriched during the transition from endo- to ecodormancy. We detected significant changes in the contents of soluble sugar, sucrose, and starch in underground buds. Additionally, three DAMs involved in starch and sucrose metabolism were identified through metabolomic analysis, including amylose, levan (a homopolysaccharide that contains fructose), and UDP-glucose. Thus, to obtain insights into the regulation of sugars, WGCNA was employed to identify DEGs with similar expression patterns related to six metabolites, including soluble sugar, sucrose, starch, UDP-glucose, amylose, and levan. DEGs with low expression (averaged FPKM <1) were filtered out, resulting in a total of 11,229 DEGs. A total of 21 distinct modules were identified with similar expression patterns (**Figure 6A**). Based on the Pearson correlation coefficients among the analyzed traits and gene modules, seven modules that were significantly associated with sugar content were identified. The purple module was positively correlated with the soluble sugar sucrose, and UDP-glucose contents (*r* = 0.786, 0.828, and 0.965, respectively) (**Figure 6B**). The red module was positively correlated with starch and amylose content (*r* = 0.79 and 0.616). The magenta module exhibited a positive relationship with the levan content (*r* = 0.826). These results suggest that the genes within these modules positively regulate the accumulation of these six sugars. Conversely, the blue module was negatively correlated with soluble sugar and levan contents (−0.721 and −0.78, respectively). The genes in the magenta, yellow, and green-yellow modules were highly negative correlated with the contents of starch, sucrose, and amylose (*r* = −0.868, −0.906, and −0.614), respectively. This suggests that these genes are involved in the negative regulation of sugar accumulation. In addition, we found no structural genes involved in starch and sucrose metabolism in the light yellow module. Consequently, genes from these six modules were selected for further analysis. KEGG enrichment analysis revealed that the DEGs were significantly enriched in starch and sucrose metabolism, and plant hormone signal transduction (**Figure 6C**).

### 3.7 Correlation analysis of DEGs and metabolites related to starch and sucrose metabolism

**Figure 7:**
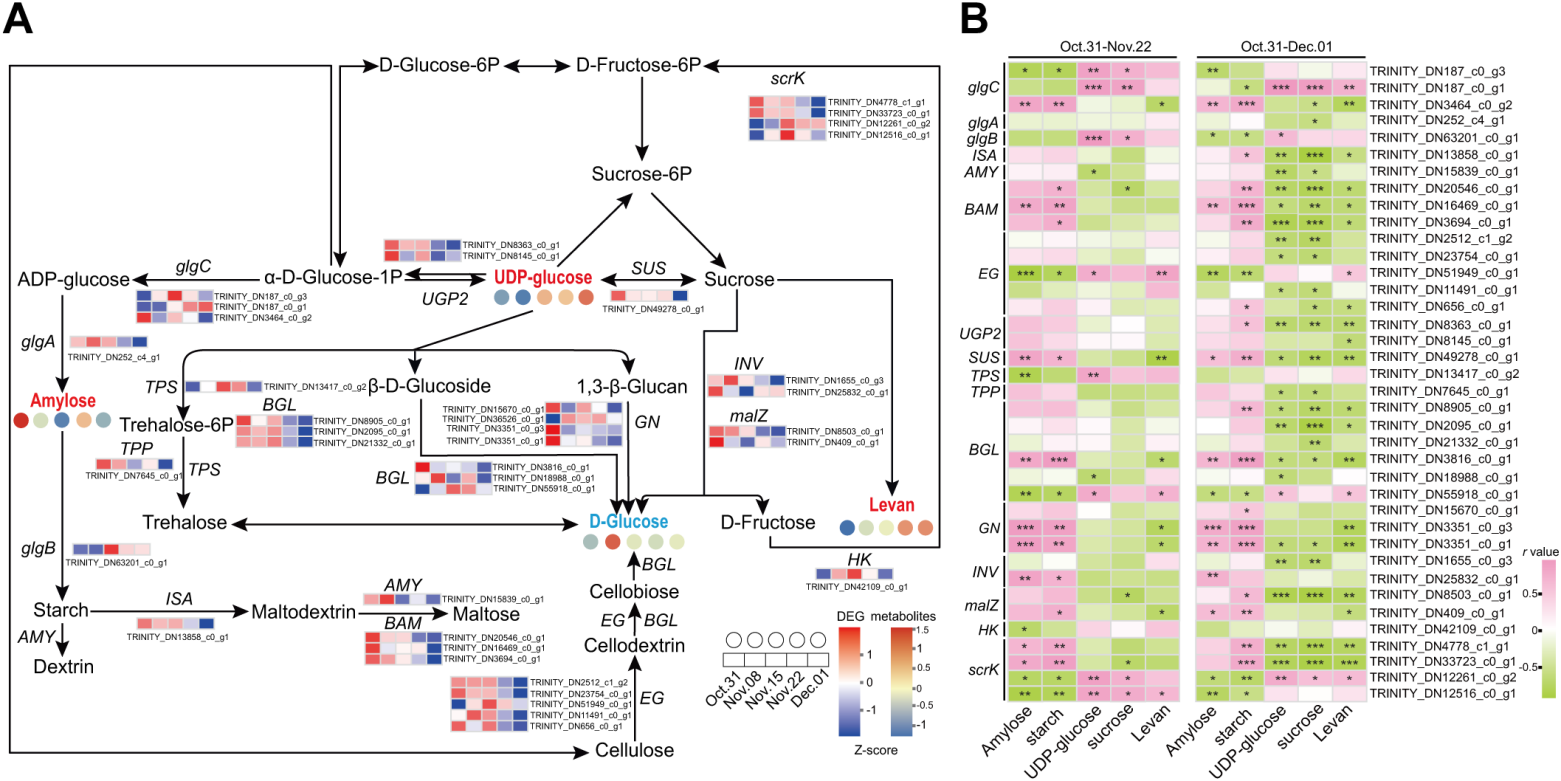
Starch and sucrose metabolism during the transition from endo- to ecodormancy in underground buds. **(A)** The heatmaps indicate the expression profiles of DEGs and metabolites involved in sucrose and starch metabolism during the transition from endo- to ecodormancy in the underground buds of AMM. **(B)** Correlation analysis between metabolites and structural genes involved in starch and sucrose metabolism. 31 October–22 November represents the endodormancy release stage, 31 October–1 December represents the transition from endo- to ecodormancy stage. The red, blue, and black fonts indicate accumulated differential metabolites, undifferentiated metabolites, and undetected metabolites, respectively. The ‘*’, ‘**’, and ‘***’ represent significant differences at the P <0.05, P <0.01, and P <0.001 levels according to Pearson’s correlation coefficient, respectively.

DEGs involved in starch and sucrose metabolism from the six modules related to sugar content were analyzed in association with the metabolites (**Figure 7**). A total of 36 DEGs involved in sucrose and starch metabolism were identified in the six modules (**Supplementary Table S10**). Metabolomic analysis revealed the presence of three DAMs involved in starch and sucrose metabolism: amylose, levan, and UDP-glucose. The *glucan endo-1,3-beta-glucosidases* (*GNs*) (TRINITY_DN3351_c0_g1, TRINITY_DN3351_c0_g3) were downregulated during the transition from endo- to ecodormancy, which was positively correlated with the content of amylose and starch, and negatively correlated with the levels of levan (**Figures 7A, B**). The expression levels of *alpha-glucosidase* (*malZ*, TRINITY_DN8503_c0_g1) and *beta-glucosidase* (*BGL18*, TRINITY_DN2095_c0_g1), which were negatively correlated with the contents of UDP-glucose and sucrose, were downregulated during endodormancy release and reached their lowest levels during ecodormancy. The level of *BGL46-like* (TRINITY_DN3816_c0_g1) decreased continuously during the transition from endo- to ecodormancy and was positively correlated with starch levels.

**Figure 8:**
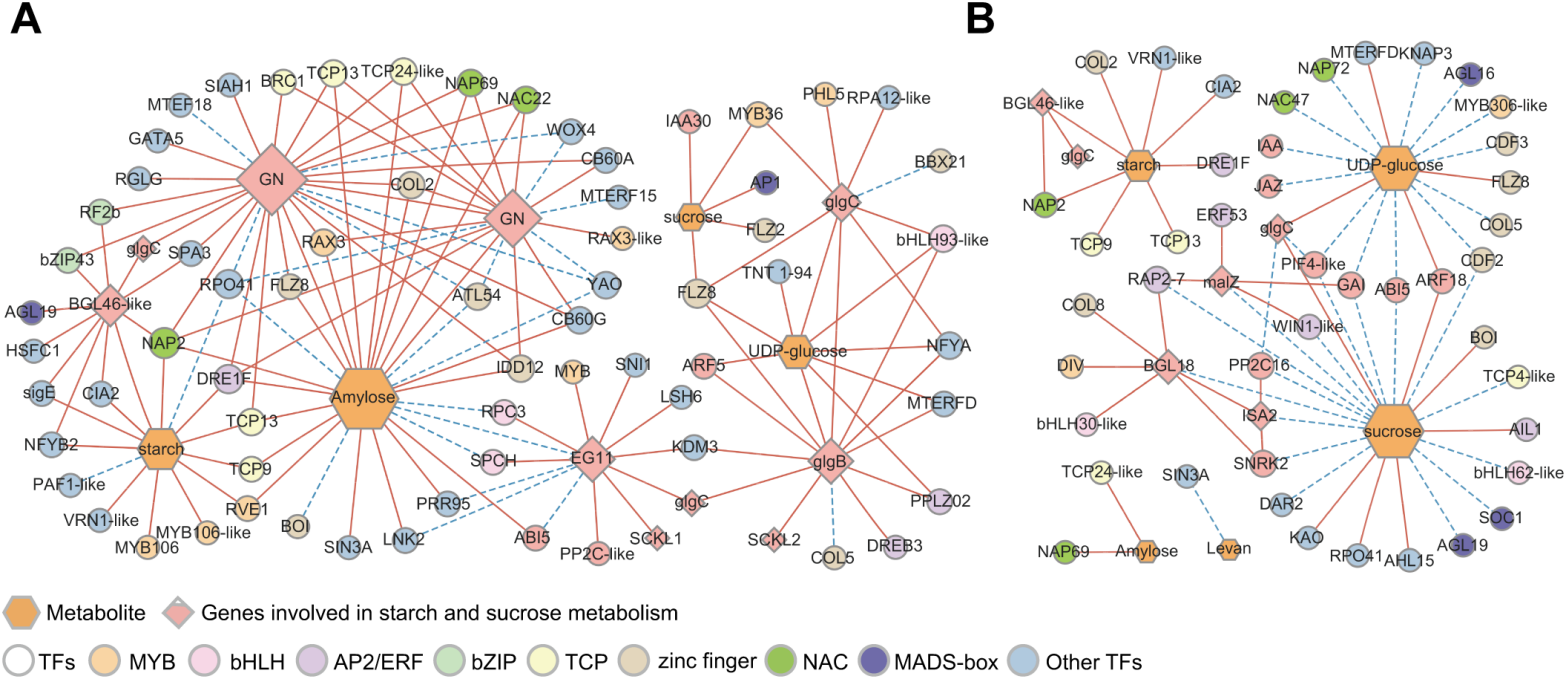
Correlation network of differential metabolites, structural genes, and TFs involved in starch and sucrose mechanism. **(A)** Co-expression networks of metabolites, structural genes, and TFs involved in starch and sucrose metabolic pathways during the process of endodormancy release. **(B)** Co-expression networks of metabolites, structural genes, and TFs involved in starch and sucrose metabolic pathways during the transition from endo- to ecodormancy. Hexagons and diamonds represent differential types of metabolites and the structural genes in starch and sucrose metabolism, respectively. TF families are represented by circles, and the color of the circles correspond to TFs of different families. The solid red line represents a positive correlation and the dashed blue line represents a negative correlation.

To better understand the key genes and TFs regulating starch and sucrose metabolism, differentially expressed TFs within the six modules were also screened, and a co-expression network of differential metabolites with DEGs and TFs was constructed (**Figure 8**). A total of 57 DEGs were highly correlated with four metabolites during endodormancy release (*r >*0.9 or <−0.9, *P <*0.05, (**Figure 8A**). The number of DEGs associated with amylose was the highest (28), and was positively correlated with amylose, including *endoglucanase* (*EG11*, TRINITY_DN51949_c0_g1) and two *GNs*. Additionally, 10 DEGs were related to UDP-glucose, and only *BGL46-like* was related to starch. The zinc finger (9), MYB (8), and TCP (5) families are likely to be TFs regulating starch and sucrose metabolism (**Figure 8A**). Furthermore, the two *GNs* were co-expressed with *COL2* (TRINITY_DN8556_c0_g1) and *DRE1F* (TRINITY_DN3958_c0_g1), and these genes were positively related to amylose. These results demonstrated that COL2 and DRE1F may regulate the expression of *GNs* and, consequently, affect the amylose content which is consistent with the decrease in *GN* expression and amylose content during dormancy release (**Figure 7B**). During the transition from endo- to ecodormancy, 51 DEGs were identified to correlate with five metabolites (*r >*0.85 or <−0.85, *P <*0.05), and most of these DEGs were negatively correlated with metabolite contents (**Figure 8B**). In addition, five structural genes were identified: *BGL18*, *BGL46-like*, *isoamylase 2* (*ISA2*, TRINITY_DN13858_c0_g1), *malZ*, and *glgC*. Compared to the endodormancy release state, *BGL18*, *ISA2*, and *malZ* may play an important role in endodormancy release. Furthermore, during the transition from endo- to ecodormancy, the zinc finger, ERF, TCP, and NAC families could be the key TFs involved in the regulation of starch and sucrose metabolism. Interestingly, we also identified that some MADS-box genes, *AP1*, *SOC1*, and *AGL19*, were all related to the concentration of sucrose, which suggested that MADS-box genes were mainly involved in regulating sucrose metabolism.

In addition, we identified genes involved in hormone signal transduction that regulates starch and sucrose metabolism. For example, a positive correlation between *ARF5* (TRINITY_DN4693_c0_g1) and *glgB* as well as UDP-glucose was identified. Additionally, there was a correlation between *glgB* and UDP-glucose (**Figure 8A**), indicating that *ARF5* may regulate the accumulation of UDP-glucose by positively regulating *glgB* expression. More hormone signal pathway-related genes were co-expressed with metabolites and structural genes related to starch and sucrose metabolism during the transition from endo- to ecodormancy (**Figure 8B**). *PIF4-like* (TRINITY_DN2672_c0_g1) negatively correlated with *glgC* and sucrose, whereas *glgC* positively correlated with sucrose. *GAI* (TRINITY_DN83684_c0_g1) also correlated with sucrose and *malZ*, and *GAI* and *malZ* were co-expressed. These findings indicate that genes related to the GA signaling pathway are involved in sucrose metabolism following endodormancy release. Overall, these results suggest that genes involved in hormone signal transduction also influence starch and sucrose metabolism by regulating the levels of UDP-glucose and sucrose.

### 3.8 Comprehensive analysis of energy metabolism and amino acid metabolism by transcriptome and metabolome

**Figure 9:**
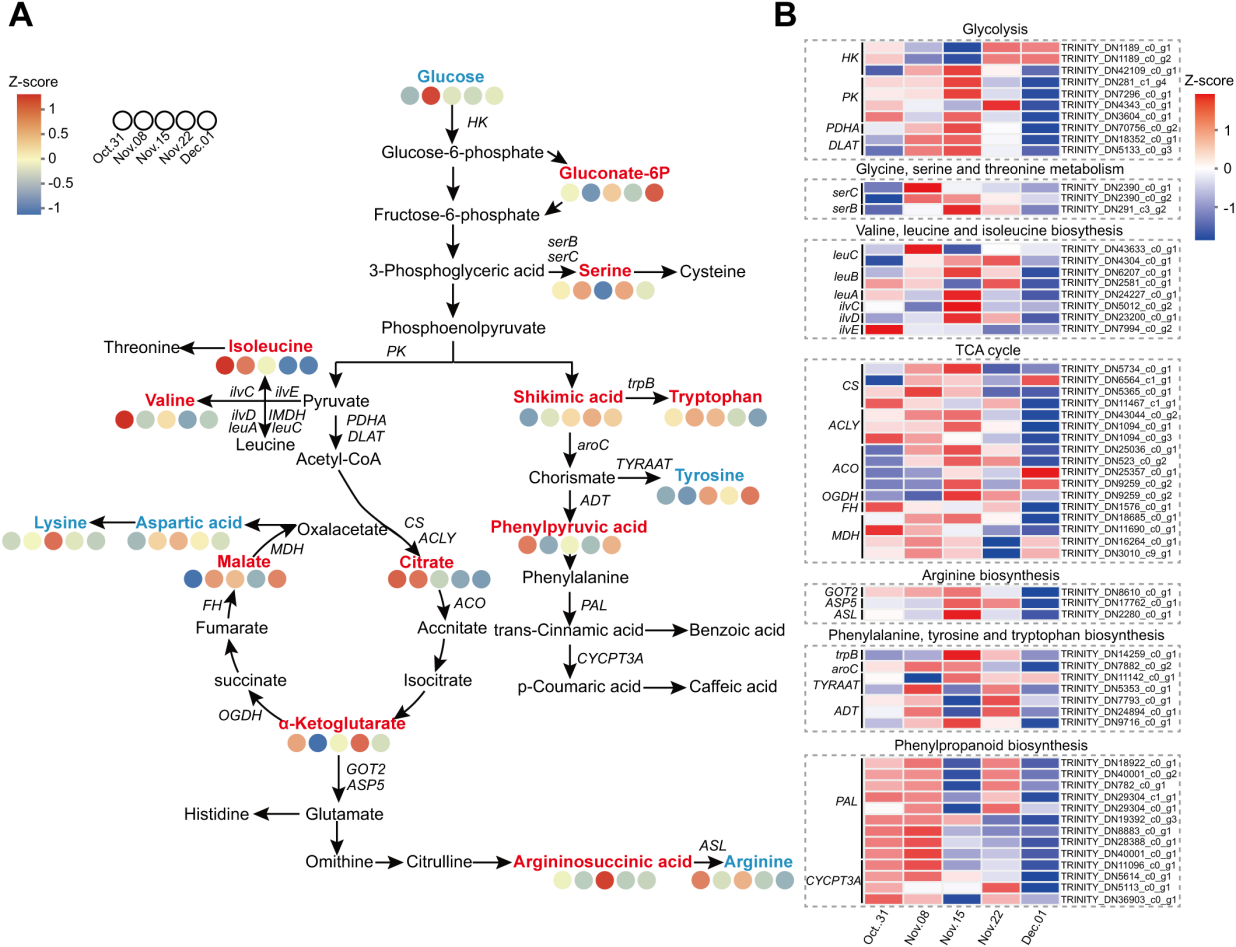
Combined analysis of the carbohydrate metabolism and amino acid metabolism pathways during the dormancy transition. **(A)** Metabolites involved in the carbohydrate metabolism and amino acid metabolism pathways. **(B)** Heatmap of the DEGs involved in the carbohydrate metabolism and amino acid metabolism pathways. The red, blue, and black fonts indicate accumulated differential metabolites, undifferentiated metabolites, and undetected metabolites, respectively.

Omics analysis showed that energy and amino acid metabolism were activated during dormancy release in AMM. Therefore, we proposed a metabolic network including glycolysis, TCA cycle, shikimic acid pathway, and amino acid metabolism to visualize carbon flow (**Figure 9**; **Supplementary Table S11**). During endodormancy release, the expression of most genes involved in glycolysis was upregulated and peaked on 15 November. This indicated that glycolysis is highly active, preparing for the complete release of endodormancy. Genes associated with the TCA cycle exhibited similar expression patterns. Most genes were upregulated during endodormancy release and downregulated during ecodormancy. On 8 November, the ketoglutarate content dropped significantly but then gradually increased until endodormancy was fully lifted. In contrast, malate levels, which are the downstream products of ketoglutarate, showed opposite trends, aligning with the patterns of gene expression. The results showed that during endodormancy release, genes associated with energy metabolism reacted quickly, resulting in active metabolic activity and providing energy for transition into an ecodormancy state. Regarding amino acid metabolism, the contents of serine and isoleucine decreased with endodormancy release, while the contents of arginine, lysine, valine, tryptophan, and tyrosine increased. The expression patterns of DEGs involved in amino acid metabolism also showed a similar trend.

## 4 Discussion

### 4.1 The combination of morphological analysis, transcriptome and metabolome provides the basis for determining the dormancy states

It is essential to have a sufficient CR for the transition from endo- to ecodormancy. We found that *Astragalus membranaceus* (Fisch.) Bge. var. *mongholicus* (Bge.) Hsiao (AMM) did not sprout completely on 31 October through the observation of several morphological indices (DFS, DBS, BPF, and DAS). The sprouting rates on 22 November reached 100% for the first time 4 weeks after transplanting, and several indices remained stable. Based on the research by Wang et al. (2022a) on the CR of *P. lactiflora*, we speculated that AMM emerged from endodormancy on 22 November. In addition, cluster analysis showed that differential metabolites accumulated significantly in underground buds collected on 31 October, 22 November, and 1 December (**Figure 2D**). These results indicate that the underground bud status of 31 October, 15 November, and 1 December may be different. Interestingly, unlike the accumulation pattern of DAMs, the number of DEGs was the highest on 15 November (**Figure 3C**). When comparing AMM that was transferred into the greenhouse on 8 November with AMM transferred on 15 November, we observed a significant increase in the BPF for the latter. However, there were no significant changes in DBS of the AMM transferred on 15 and 22 November. We speculate that, at this time, AMM may be in the early stages of fully breaking endodormancy, as it appears to have reached the minimum CRs for normal growth. This stage triggers the expression of a large number of genes, prepares the plant to completely break dormancy, and promotes various biosynthetic, degradative, and metabolic processes (Zhang et al., 2021b). In summary, it is determined that it is in endodormancy stage on 31 October, and 15–22 November is the stage when AMM underground buds completely break endodormancy. This study provides guidance for determining the period of dormancy release of AMM and clarifying the CR for dormancy release.

### 4.2 Endogenous hormones are actively involved in the dormancy transition of underground buds of AMM

The dormancy of perennial plants is primarily regulated by hormones that participate in the formation of a complex regulatory network during dormancy release (Liu and Sherif, 2019; Pan et al., 2021). IAA is an important hormone for plant growth and development and is involved in the regulation of bud dormancy (Hao et al., 2019). In this study, the IAA levels in underground buds were significantly higher than those of the other three hormones (**Figure 1A**), and a large number of DEGs related to IAA signaling pathways were identified (**Figure 4**). Similar findings have been reported in the Chinese fir (*C. lanceolata*) (Qiu et al., 2013) and plum (*Prunus mume*) (Zhang et al., 2018). Furthermore, co-expression network analysis showed that DEGs in the auxin signaling pathway exhibited greater connectivity than genes related to other hormone signaling pathways during endodormancy release (**Figure 5A**). These results highlight the key role of IAA in the endodormancy release of AMM underground buds. In most species, ABA and GA are considered key regulators of bud dormancy (Li et al., 2020; Zhang et al., 2018; Gao et al., 2023). The levels of ABA and GA also changed during underground bud dormancy release in AMM. Contrary to the common belief that a reduction in ABA content triggers dormancy release, our results showed that ABA levels remained high during endodormancy release, as observed in the upregulation of the biosynthesis gene *NCEDs* and the low level of the degradation gene *CYP707A2* (**Supplementary Figure S3**), which was consistent with the results of Wang et al. (2019). This indicates that high levels of ABA may positively influence release from bud dormancy. However, further research is needed to verify the role of ABA in this process in AMM underground buds. Similar regulatory patterns were observed for GA synthesis genes *GA20ox* and *GA3ox*, and degradation gene *GA2ox* during the process, which allowed GA3 to maintain an upward trend throughout the release of endodormancy, which is consistent with the results observed in tree peony (Yuan et al., 2024).

In perennial plants, ABA, GA, IAA, and CK interactions regulate bud dormancy (Lv et al., 2018; Tosetti et al., 2021). The combined application of CTK and GA can effectively break the dormancy of tea buds within a short period (Thirugnanasambantham et al., 2020). This study found that IAA, ABA, GA, and CTK levels exhibited consistent trends during endodormancy release (**Figure 1A**). Additionally, co-expression network analysis revealed that genes associated with IAA, ABA, GA, and CTK signaling pathways were co-expressed during endodormancy release, with IAA signaling pathway genes showing stronger connectivity (**Figure 5**). In summary, we speculated that these hormones may form a synergistic network in regulating endodormancy release, with IAA potentially playing a leading role in this process. However, further research is needed to fully understand how these four hormones work together to regulate dormancy.

### 4.3 Carbohydrates participate in the regulation of the bud dormancy transition of AMM

It is well established that carbohydrates are closely related to dormancy (Gillespie and Volaire, 2017). Perennials’ dormant buds appear to be in an inactive state with limited metabolic activity, and reconfiguration of carbohydrate metabolism is associated with dormancy transition. The decrease in starch content and increase in soluble sugars trigger dormancy release (Marković et al., 2020). In our study, there was a significant decrease in the starch content at the onset of endodormancy release, followed by a gradual increase before dormancy was fully released. Subsequently, the starch content began to decline again, which was closely associated with the dormancy stage (**Figure 2B**). In addition, the level of levan increased during endodormancy release and reached a high level when endodormancy was released (**Figure 1A**), indicating that starch and levan could serve as potential markers for distinguishing the stages of dormancy (Ohanenye et al., 2019). As starch levels decreased, soluble sugar levels increased until ecodormancy was achieved. This observation aligns with metabolomic analyses showing that significantly abundant DAMs are enriched in starch and sucrose metabolism during ecodormancy. In addition, genes related to starch and sucrose metabolism were highly expressed during endodormancy release. We speculated that asynchrony in the expression of genes involved in starch and sucrose metabolism and metabolite accumulation may contribute to the promotion of endodormancy release. Genes involved in the synthesis and degradation of carbohydrates such as starch and sucrose were actively expressed. This activity was intended to provide adequate resources for respiration and energy metabolism. Therefore, there were few significant changes in metabolite content until endodormancy release. During the ecodormancy stage, metabolite levels peak in preparation for the subsequent regeneration stage (Zhang et al., 2020). However, more evidence is required to support this hypothesis.

Co-expression network analysis showed that *GNs*, *EG*, *BGL*, *glgB*, and *glgC* were associated with the differential metabolism of sugars during endodormancy release. We showed that *GN* plays an important role in starch degradation and the response to biotic and abiotic stresses (Nakatani et al., 2010). In our study, *GN* expression levels were highly correlated with starch and amylose during endodormancy release, and they had the highest levels of TF co-expression (**Figure 7B**). However, this correlation no longer exists following endodormancy release. We hypothesized that *GNs* may be important in regulating starch degradation during endodormancy release (Gao et al., 2021). Zeng et al. (2021) found that the decreased expression of *BGL* may be related to a decrease in sucrose content. In this study, most *BGLs* maintained a high level during endodormancy release, which was positively correlated with the sucrose content. Similar findings have also been reported for rhubarb (Wojtania et al., 2022) and *P. lactiflora* (Zhang et al., 2021a). In conclusion, *GNs* and *BGL* may play key roles in the release of endodormancy by regulating starch and sucrose metabolism.

Some studies have confirmed a relationship between carbohydrates and hormones during dormancy release (Wang et al., 2022a). This study found that the trends of the four hormone levels changed in the opposite direction to that of the starch content. Additionally, *SNRK2* in the ABA signaling pathway was correlated with sucrose content during the transition from endo- to ecodormancy (**Figure 8B**). Additionally, the trend of the expression patterns of most *SNRK2* genes was similar to that of the changes in ABA content. It has been speculated that the crosstalk between sucrose and ABA is mediated by *SNRK2*. *AMY*, which plays a key role in dormancy, also regulates the interaction between hormones and sugars (Xie et al., 2007). GA upregulates *AMY* expression through *GAMYB*, thereby promoting starch degradation (Li et al., 2020). The variation in GA_3_ levels in the underground buds of AMM during endodormancy release was opposite to the change in starch content. The expression pattern of *GAMYB* (TRINITY_DN15272_c0_g1) was consistent with GA_3_ levels, and *GAMYB* was positively and negatively correlated with GA_3_ and starch content, respectively (**Supplementary Table S12**). Therefore, we hypothesized that GA participates in starch degradation by GAMYB during the transition from endo- to ecodormancy (Chao et al., 2006).

The physiological activities involved in the transition to bud dormancy require significant energy and material resources, primarily obtained from the breakdown of stored substances. Metabolomic analysis revealed that, during the ecological dormancy stage, DAMs were notably enriched in starch and sucrose metabolism. This indicated that soluble sugars gradually accumulated, suggesting that the increase in soluble sugar content may fulfill the energy and material requirements for bud regrowth. In this study, most of the DEGs involved in glycolysis and the TCA cycle were upregulated during endodormancy release (**Figure 9**). This finding aligns with previous observations made during bud dormancy in peaches (Tang et al., 2019) and tree peonies (Zhang et al., 2020). This indicated that the activation of energy metabolism occurs through the upregulation of these genes during the endodormancy release stage, allowing them to serve as the primary energy source. Glycolysis and TCA cycle can also provide precursors for amino acid synthesis during budbreak. Metabolomic analysis showed that DAMs were significantly enriched in amino acid metabolism during the transition from endo- to ecodormancy, which is similar to the findings of Cheng et al. (2023). These results indicate that AMM may respond to endodormancy release through high levels of amino acids and their derivatives. Therefore, we speculated that amino acid metabolism might be necessary for endodormancy release in AMM.

### 4.4 The role of TFs in the release of dormancy

An increasing number of studies have demonstrated that the molecular mechanisms regulating dormancy are mediated by complex genetic networks composed of many TFs, such as WRKY, MYB, bZIP, NAC, and bHLH (Wang et al., 2018; Nakatani et al., 2010). Based on the co-expression network analysis, it was found that the dormancy of AMM underground buds was related to some key TF families, including WRKY, AP2/ERF, MYB, bHLH, zinc finger, HD-ZIP, and MADS-box. Interestingly, WRKY families were not screened for TFs regulating starch and sucrose metabolism, but Fan et al. (2021) found that WRKY32 and WRKY71 were closely related to sucrose metabolism during the dormancy transition of lily bulbs, which may vary from species to species. Moreover, our studies have shown that *DREB1*, *DREB2*, and *DREB3* are co-expressed with genes associated with the IAA and ABA signaling pathways during endodormancy release. This result implies a significant role of DREB in the endodormancy release process, which aligns with the findings of Artlip et al. (2019). Genes encoding MADS-boxes are highly correlated with bud dormancy in many species (Wu et al., 2017), among which *SOC1* has been proven to be crucial for breaking bud dormancy (Gómez-Soto et al., 2021). In this study, *SOC1* was highly associated with *PIF4-like* proteins and sucrose during the transition from endo- to ecodormancy, indicating that *SOC1* may be essential for regulating dormancy by simultaneously controlling the GA signaling pathway and sucrose accumulation (Yang et al., 2021). In addition, the flowering regulator AP1 is also involved in the expression of IAA signaling pathway genes and sucrose accumulation during dormancy release. These results suggest that the regulatory network mediated by TFs, including WRKY, AP2/ERF, bHLH, zinc finger, and MADS-box, has a potential function in regulating dormancy release in underground buds of AMM.

## 5 Conclusion

**Figure 10:**
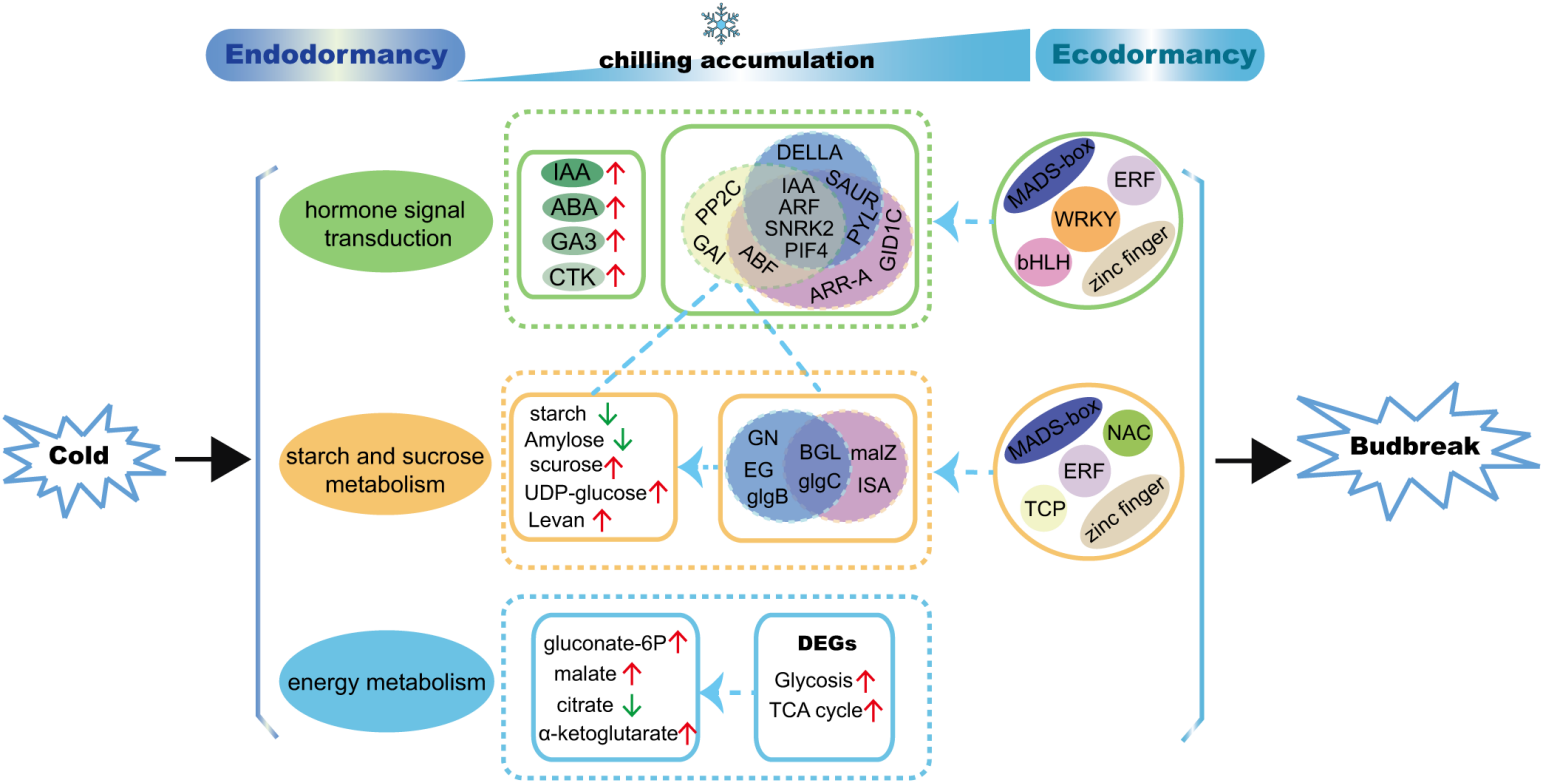
A proposed model for regulating the transition from endo- to ecodormancy in underground buds of AMM induced by chilling. The blue circles represent genes that may play a role in the process of endodormancy release, the purple circles represent genes that may be active following endodormancy release, the yellow circles represent genes that are co-expressed with genes and metabolites related to starch and sucrose metabolism.

In this study, the regulation mechanism of underground bud dormancy of *A. membranaceus* (Fisch.) Bge. var. *mongholicus* (Bge.) Hsiao (AMM) was studied by a multi-omics approach for the first time. We comprehensively analyzed the metabolic and transcriptome data of the underground buds of one-year-old AMM at five successive dormancy stages and proposed a model for regulating the dormancy transition of underground buds of AMM induced by chilling (**Figure 10**). IAA may be a key hormone in the regulation of endodormancy release. Genes related to the IAA, ABA, and GA signaling pathways (*IAA*, *ARF*, *SNRK2*, and *PIF4*) were involved in regulating starch and sucrose metabolism during the transition from endo- to eco-dormancy. In addition to starch and sucrose, levan may also be used as a marker of dormancy. Moreover, the structural genes, including *GN*, *EG*, and *glgB*, for starch and sucrose metabolism may be associated with endodormancy release, whereas *ISA* and *malZ* are involved in regulating starch and sucrose metabolism following endodormancy release. Through co-expression network analysis, we found that TFs of the ERF, MYB, bHLH, zinc finger, and MADS-box families may control bud dormancy by regulating the expression of genes involved in hormone signal transduction and carbohydrate metabolism. Although the WRKY family only regulates hormone signal transduction, the interactions among molecules, cells, and metabolism need to be further explored. We hope that our study can contribute to a better understanding of the regulatory mechanism of the transition from endo- to ecodormancy in AMM and provide a new strategy for breaking dormancy and shortening the breeding time in advance.

## Data Availability

The datasets presented in this study can be found in online repositories. The names of the repository/repositories and accession number(s) can be found in the article/**Supplementary Material.**
