# Supplementary material for: Integrated metabolome and transcriptome analysis reveals potential mechanism during the bud dormancy transition of Astragalus membranaceus (Fisch.) Bge. var. mongholicus (Bge.) Hsiao
Source: Front Plant Sci. 2025 Jan 21;15:1483538. doi: 10.3389/fpls.2024.1483538 (PMC11790638; doi:10.3389/fpls.2024.1483538)
Supplement: Supplementary file 2 [file DataSheet1.docx]

Supplementary Material

**
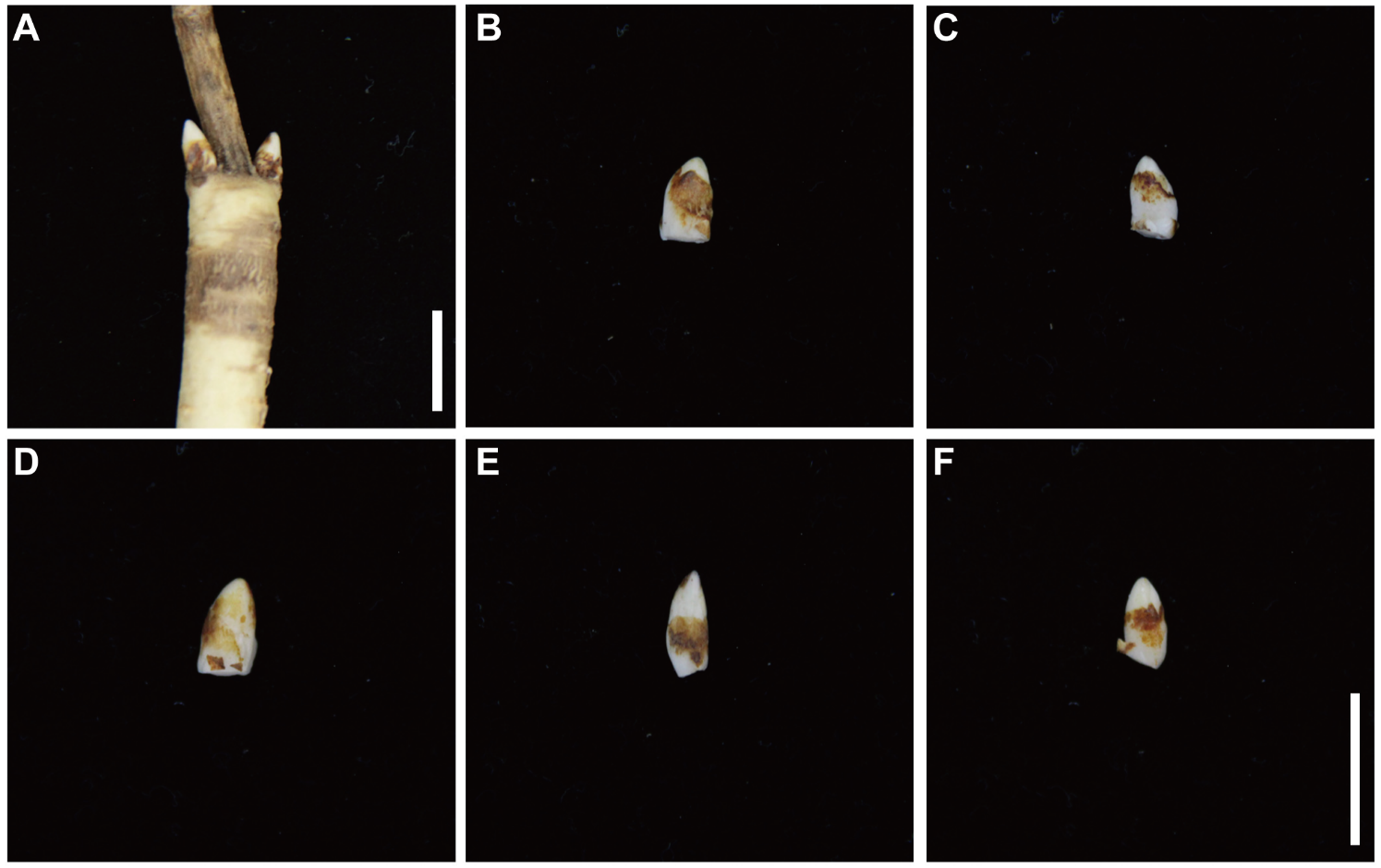
**

**Figure S1.** Underground bud morphology of annual AMM. **(A)** Underground buds growing on the crow. **(B-F)** refer to underground buds collected on October 31, November 08, November 15, November 22, and December 01, respectively. Bar = 1 cm.


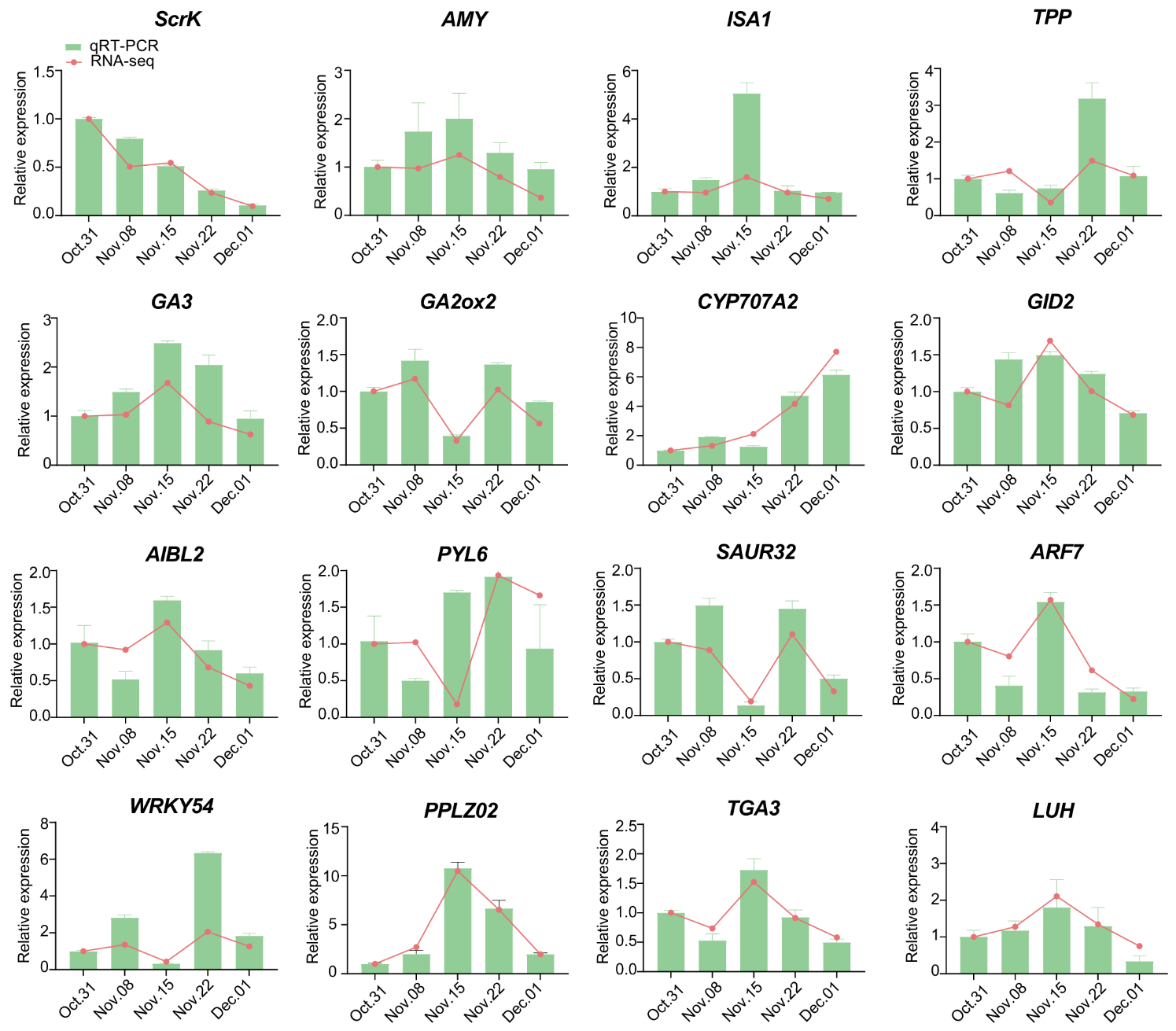


**Figure S2.** qRT-PCR verification of related genes in transcriptome. The green columns indicate the relative expression levels detected by qRT-PCR using the 2^−ΔΔCT^ method. The red lines represent the fold changes in the RNA-seq data. The relative expression level of each gene is expressed as a fold change relative to the control (Oct. 31). These values represent the means ± SD of three biological replicates.

**
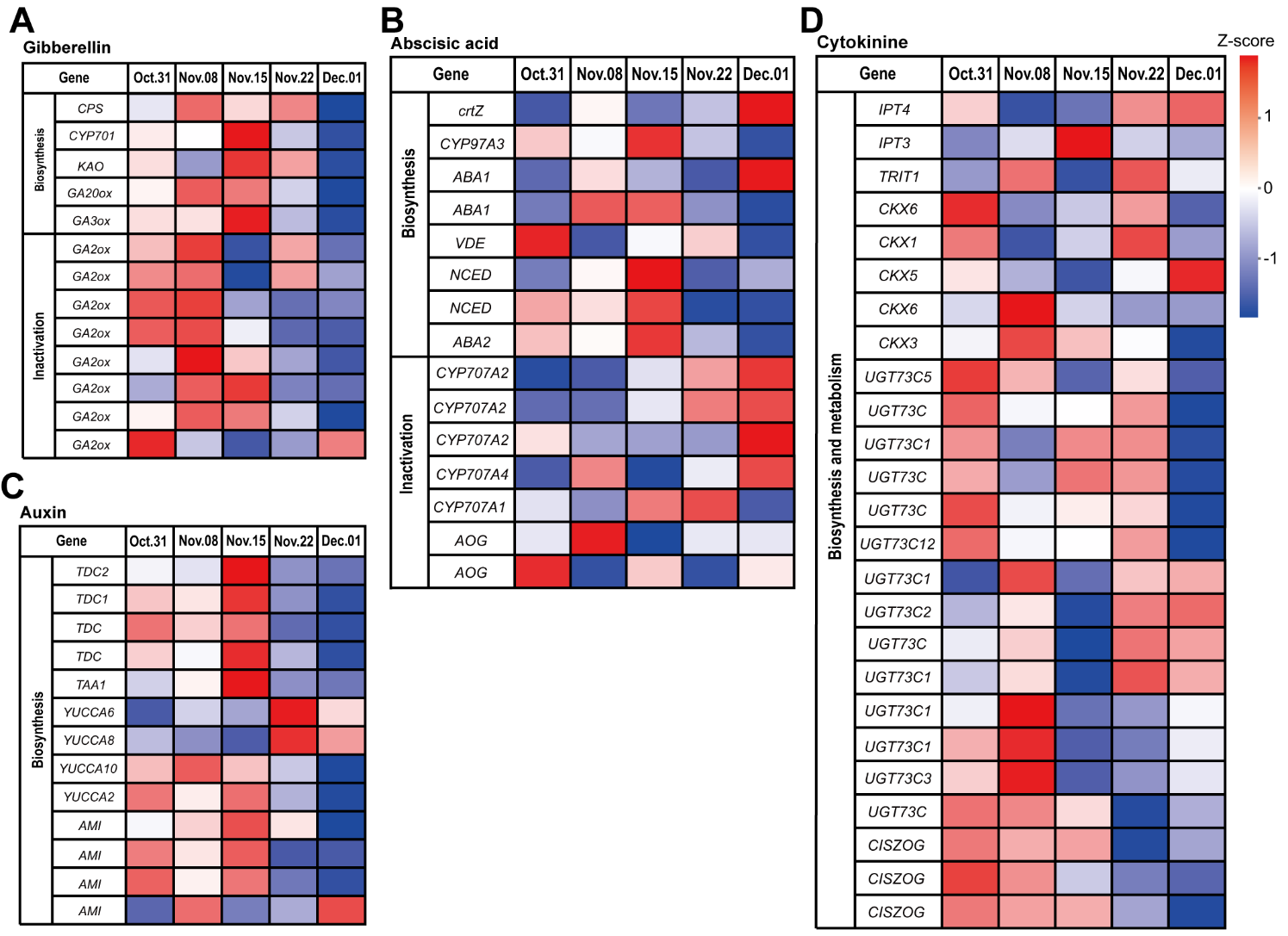
**

**Figure S3.** Analysis of phytohormones during the transition from endo- to ecodormancy in AMM. The relative expression of GA **(A)**, ABA **(B)**, IAA **(C)**, and CTK **(D)** biosynthesis genes during different dormant stages of AMM. Error bars in the line graph indicate the mean ± SD of three independent biological replicates. The letters indicate significant differences based on Duncan′s multiple comparisons from one-way ANOVA, *P*＜0.05.
